# Predicting individual differences in digital alcohol intervention effectiveness through multimodal data

**DOI:** 10.1038/s41746-026-02356-4

**Published:** 2026-01-27

**Authors:** Magdalena Fuchs, Zachary M. Boyd, Alice Schwarze, Danielle Cosme, Ovidia Stanoi, Yoona Kang, Tobias Kowatsch, Florian von Wangenheim, Dani S. Bassett, Kevin N. Ochsner, David M. Lydon-Staley, Emily B. Falk, Peter J. Mucha, Mia Jovanova

**Affiliations:** 1 Centre for Digital Health Interventions, Department of Management, Technology and Economics, ETH Zürich, Zürich, Switzerland; 2 Department of Mathematics, Brigham Young University, Provo, UT USA; 3 Office of Artificial Intelligence Policy, Utah Department of Commerce, Salt Lake City, UT USA; 4 Annenberg School for Communication, University of Pennsylvania, Philadelphia, PA USA; 5 Annenberg Public Policy Center, University of Pennsylvania, Philadelphia, PA USA; 6 Department of Psychology, Rutgers, The State University of New Jersey, New Jersey, NJ USA; 7 Centre for Digital Health Interventions, Institute for Implementation Science in Health Care, University of Zürich, Zürich, Switzerland; 8 Centre for Digital Health Interventions, School of Medicine, University of St. Gallen, St. Gallen, Switzerland; 9 Department of Bioengineering, University of Pennsylvania, Philadelphia, PA USA; 10 Department of Electrical & Systems Engineering, University of Pennsylvania, Philadelphia, PA USA; 11 Department of Neurology, University of Pennsylvania, Philadelphia, PA USA; 12 Department of Psychiatry, University of Pennsylvania, Philadelphia, PA USA; 13 Department of Physics & Astronomy, University of Pennsylvania, PA Philadelphia, USA; 14 The Santa Fe Institute, Santa Fe, NM USA; 15 Department of Psychology, Columbia University, New York City, NY USA; 16 Leonard Davis Institute of Health Economics, University of Pennsylvania, Philadelphia, PA USA; 17 Department of Psychology, University of Pennsylvania, Philadelphia, PA USA; 18 Penn Center for Science, Sustainability, and the Media, University of Pennsylvania, Philadelphia, PA USA; 19 Wharton Marketing Department, University of Pennsylvania, Philadelphia, PA USA; 20 Wharton Operations, Information and Decisions Department, University of Pennsylvania, Philadelphia, PA USA; 21 Department of Mathematics, Dartmouth College, Hanover, NH USA

**Keywords:** Health care, Psychology, Psychology

## Abstract

Digital interventions can change behaviors like alcohol use, but effectiveness varies widely across individuals. Accurately identifying non-responders—i.e., those least (vs. most) likely to change their behavior—before intervention delivery is difficult. Individual intervention effectiveness predictions from prior studies perform only slightly above chance (e.g., AUC ≈0.60; balanced accuracy ≈0.60). We present a novel approach integrating multimodal data across theory-driven domains—including psychological assessments, social network data, and neural responses to alcohol cues—to make ex-ante predictions about the effectiveness of smartphone-delivered alcohol interventions targeting psychological distancing in young adults (Study 1: *N* = 67; Study 2: *N* = 114). Demonstrating the feasibility of this approach, random forest models predicted individual differences in intervention effectiveness (Study 1: balanced accuracy = 0.71, 95% CI: 0.69–0.73, *p* = .020; AUC = 0.87, 95% CI: 0.85–0.88, *p* = .020) and replicated in a an external test sample (Study 2, balanced accuracy = 0.68; AUC = 0.68, 95% CI: 0.54–0.82), meeting clinical-utility thresholds from prior digital health studies (balanced accuracy = 0.67; correctly classifying (non)responders 67% of the time). Interventions were most effective for participants who perceived their peers as moderate but frequent drinkers. Peer drinking perceptions may serve as a low-burden indicator to support early identification of non-responders in preventive alcohol interventions among young adults. Future work can apply and extend the multimodal approach developed here for adaptive tailoring of digital behavior change interventions in real-world settings.

## Introduction

Alcohol use remains a significant global public health challenge, contributing to leading causes of mortality and disability, such as cardiovascular disease, cancers, and liver disease^1,2^. Drinking habits developed in young adulthood often persist into later adulthood, increasing long-term risk of alcohol-related harms^3^. Consequently, the development of effective interventions to prevent unhealthy drinking habits among young adults remains a public health priority^4^. Digital health interventions (DHIs)—which typically use mobile technologies such as smartphones to support behavior change^5^—are increasingly popular strategies to target drinking in daily life^6–9^.

Among the various techniques embedded within DHIs, psychological distancing—which aims to create mental space from triggers such as alcohol cues^10^—has shown promise to regulate appetitive responses to alcohol^11,12^. However, the effectiveness of psychological distancing-based DHIs varies between individuals; although DHIs can help some individuals to reduce their drinking, they have little or no effect on others^8,13^. This heterogeneity raises important questions about who is more (vs. less) likely to respond, or change their drinking behavior in response to psychological distancing DHIs^5,14^. Identifying likely responders (i.e., those who reduce their drinking) from non-responders (i.e., those who do not change, or possibly increase their drinking) is a first step to assess the scalability of existing interventions^15^ and to tailor more effective DHIs to individual psychological, behavioral, and contextual characteristics^16–18^.

Which individual-level factors predict who is more (vs. less) likely to respond to psychological distancing DHIs? A wide range of individual-level factors have been proposed to influence drinking behaviors and responsiveness to psychological distancing interventions among young adults. Health psychology and health communication literatures highlight the role of motivations, attitudes, self-efficacy, and intentions as determinants of alcohol use^19,20^, and underscore the influence of peer drinking behaviors—both perceived and objective—on drinking patterns^21–23^. Clinical psychology research highlights traits such as impulsivity, affect regulation, and attentional control, which can alter how individuals react to alcohol cues and, in turn, respond to interventions^24–26^. Neuroscience further points to differences in reward, regulatory, and social-cognitive brain systems that shape how people process alcohol cues and engage with psychological distancing-based strategies^27–30^. Although each of these domains identifies plausible predictors of DHI response, they are typically examined separately^31,32^. This siloed approach limits our ability to understand how these diverse candidate factors predict individual differences in DHI effectiveness.

Identifying which individuals are least vs. most likely to change their behavior, and which baseline factors are most predictive of behavior change, is fundamental for scalable DHI deployment^33^, motivating recent efforts to use machine learning (ML) methods to predict intervention outcomes. However, despite growing interest in ML-based DHI evaluation^34–36^, it remains unclear how accurately behavior change can be predicted in advance (i.e., before the start of the intervention period), and which theory-driven baseline factors are most predictive. Existing work has largely focused on predicting intervention adherence—i.e., how likely participants are to engage with intervention content—rather than intervention-driven behavior change^17,34,35^. When behavior change is the outcome, DHI evaluation studies often rely on concurrent intervention engagement metrics for prediction (e.g., app logins or clicks)^18,37,38^, use limited feature sets as predictors^17,18^, and lack external testing on new, unseen data^17,18,38^. For example, one study examining DHI effectiveness (defined as program completion and goal achievement) found that app usage during the first three days predicted reductions in alcohol use over the next four months (AUC = 0.71), but the model was not externally tested on held-out participants^18^, raising concerns about inflated performance. Another study predicting individual differences in DHI effectiveness for depression and anxiety symptoms used baseline mental-health and work-related questionnaires, but achieved only modest accuracy (AUC = 0.60, balanced accuracy = 0.60)^16^—meaning it correctly classified responders vs. non-responders to the intervention 60% of the time. Such models fall short of proposed clinical benchmarks for adaptive DHI tailoring, which suggest correctly identifying responders and non-responders at least two-thirds of the time (balanced accuracy = 0.67)^37,38^. Overall, these findings indicate that predicting non-responders (vs. responders) before an intervention remains challenging, with prior models performing only slightly above chance.

Building on this work, we took a novel, interdisciplinary approach by integrating multiple theoretically grounded domains—spanning psychological, behavioral, neuroimaging, social network, and demographic factors^39–42^—to predict individual differences in response to psychological distancing alcohol DHIs in young adults. We investigated two primary research questions. First, to what extent can psychological, social, behavioral, neuroimaging, and demographic factors, collected at baseline, predict individual differences in DHI effectiveness (i.e., reductions in alcohol use occasions), and which factors are most predictive (RQ1)? Second, does a model trained on the most predictive factors replicate in an external test set of participants who completed a parallel intervention protocol (RQ2)? Thus, our study primarily aims to classify DHI responders (from non-responders) across two different student cohorts. While follow-up feature-importance analyses can generate hypotheses about underlying behavioral mechanisms, our models are primarily designed for prognostic prediction rather than causal or mechanistic explanation of individual differences in intervention effects.

To examine these questions, we conducted a secondary analysis on data collected from two cohorts of the SHINE project^43^, in which two samples of college students who are social drinkers (Study 1: *N* = 67; Study 2: *N* = 114) completed 28-day psychological distancing interventions delivered via smartphones. In both studies, participants were assigned to one of two psychological distancing intervention conditions or to a control condition. Our analyses focus on participants in the intervention conditions, who received either a mindfulness or perspective-taking intervention strategy. The “mindfulness” strategy encouraged participants to notice and accept their own thoughts and feelings related to alcohol (e.g., “If you are around alcohol today, react mindfully—notice, acknowledge, and accept the thoughts and feelings you have”). The “perspective-taking” strategy encouraged participants to consider how a peer they considered to be a low drinker might think or feel in the same situation (e.g., “If you are around alcohol today, imagine how [peer name] would react—try to imagine the thoughts and feelings that [peer name] would have”)^44^. Both strategies were delivered through twice-a-day text messages in a within-person design, alternating between two weeks of intervention (active weeks) and two weeks with control texts, which encouraged participants to respond naturally to alcohol (inactive weeks), allowing for within-person assessment in behavior change over time. For more details on intervention design, refer to the SHINE protocol (ref. ^43^). All analyses in the main manuscript pool both psychological distancing strategies (perspective-taking and mindfulness) into a single intervention group due to sample size constraints. Consistent with our prior report on average intervention effects^12^, we define DHI effectiveness as a reduction in drinking occasions during “on” (intervention) weeks vs. “off” (inactive) weeks. Guided by theoretical perspectives (detailed in Supplement 1), we evaluated how factors across six theory-driven domains predicted variation in intervention response among held-out, unseen, participants. These domains included: (1) alcohol use attitudes, intentions, and past drinking behaviors^39,40^; (2) psychological traits such as anxiety^24^, impulsivity^45^, and susceptibility to peer influence^46^; (3) sociometric centrality measures from existing peer groups^21^; (4) neural responses to alcohol-related cues^47^, social cues^41,42^, and resting-state activity^41,42^; (5) subjective perceptions of peer drinking^23,48^; and (6) demographics. Supplement 3 details the specific measures across all domains. We tested four machine-learning classifiers with varying model complexity: A random forest model, penalized logistic regression (Elastic Net), linear support vector machine (SVM), and radial basis function SVM. Model performance was estimated using nested cross-validation, with an inner loop for hyperparameter tuning and an outer loop for out-of-sample evaluation to prevent overfitting. The best-performing model for each model type was tested on an external, non-overlapping test set and evaluated using AUC, F1 score, and balanced accuracy^49^. AUC measures the model’s ability to discriminate between classes (responders vs non-responders), F1 score emphasizes correct classification of positives (responders), and balanced accuracy averages how well a model correctly identifies both responders (true positives) and non‑responders (true negatives). Importantly, balanced accuracy weights sensitivity and specificity equally, making it preferable to standard accuracy for imbalanced data^49^. Feature importance was assessed using SHAP values^50^, which estimate each candidate factor’s contribution to the model’s predictions.

## Results

### Sample descriptives

Participants included college students who are members of on-campus social groups (e.g., sports teams, sororities/fraternities, performing arts groups, and technology clubs) from two university campuses in the Northeastern United States, recruited as part of the SHINE project^43^. See Methods, Data/Sample sections and ref. ^43^ for more details on participant eligibility and recruitment. Study 1 sample included 67 participants (41 women, 26 men, and 0 non-binary), of whom 37 (55.2%) were assigned to the mindfulness strategy and 30 (45.8%) to the perspective-taking strategy. Study 2 included 114 participants (91 women, 23 men, and 0 non-binary), of whom 58 (50.9%) were assigned to mindfulness and 56 (49.1%) to perspective-taking. Participants in Study 1 were 20.5 years old on average (SD = 1.76; range = 18–28) and identified as white (55.2%), Asian (31.3%), Black/African American (3.0%), Latino/a/x (3.0%), and multiracial (7.5%). Participants in Study 2 were 20.2 years old on average (SD = 1.26; range = 18–28) and identified as white (38.6%), Asian (35.1%), Black/African American (10.5%), Latino/a/x (5.3%), multiracial (7.2%), and missing information (1.8%). We observed high adherence in both studies (Study 1: *M* = 90.25% of alcohol prompts completed, SD = 12.62%; Study 2: *M* = 82.07% of alcohol prompts completed, SD = 25.64%). In Study 1, of the 67 participants in the intervention conditions, 37 (55.2%) were assigned to the mindfulness strategy, and 30 (45.8%) received the perspective-taking strategy. In Study 2, of the 114 participants in the intervention conditions, 58 (50.9%) were assigned to mindfulness and 56 (49.1%) to perspective-taking. For additional descriptives by intervention strategy, refer to Supplementary Table S2. Overall, we observed no significant baseline differences in age, gender, race/ethnicity, baseline drinking amount or frequency, and adherence rates (mindfulness: 91% vs. perspective-taking 90%; all *p* > 0.5) between participants assigned to the intervention sub-groups.

With respect to changes in alcohol use, 22% of participants in Study 1 and 12% in Study 2 reduced their drinking by more than one occasion between active and inactive weeks. Intervention responsiveness—defined as an average reduction of more than one drinking occasion per week—was similar across strategies. In Study 1, 27% of mindfulness participants and 17% of perspective-taking participants were responsive; in Study 2, 13.8 and 10.7%, responded, respectively. Adherence rates (percentage of alcohol prompts completed by a participant) did not differ significantly between responders and non-responders (Study 1: responders *M* = 93%, SD = 1.86%; non-responders *M* = 95%, SD = 13.42%; Mann–Whitney *U* = 368.50, *p* = 0.749; Study 2: responders *M* = 92%, SD = 14.43%; non-responders *M* = 93%, SD = 26.94%; Mann–Whitney *U* = 1242.00, *p* = 0.876). While qualitatively we observed greater variability in intervention adherence among non-responders, including several outliers with very low adherence (<10% of prompts completed), we found no association between response rates and intervention effectiveness in either study (*p* > 0.05). Responders tended to report higher baseline drinking frequency and more frequent drinking during inactive weeks as part of the intervention protocol (vs. non-responders), as shown in Supplementary Table S3. However, baseline drinking did not reliably predict intervention effectiveness in cross-validation (balanced accuracy = 0.54), indicating that differences in intervention effectiveness were not simply a function of baseline drinking behavior. Sample characteristics are reported in Supplementary Table S3 and Supplementary Table S1. Supplement 7, Tables S12, S13, and Figs. S9, S10 present drinking behavior (frequency and amount) during the intervention period by responder status and intervention week (active/inactive).

### Predicting individual differences in DHI effectiveness

To address RQ1—to what extent can we predict individual differences in DHI effectiveness and which factors contribute most—we evaluated four machine-learning classifiers that distinguish intervention responders from non-responders. Random forest and penalized logistic regression (Elastic Net) results are presented in the main manuscript. Linear and radial SVM results are provided in Supplement 6. Logistic regression and random forest capture linear and nonlinear effects, respectively, whereas linear and radial SVMs offer alternative approaches based on separating hyperplanes. Comparing multiple model types allowed us to test the robustness of our findings across methods with differing assumptions and across varying levels of complexity^51^.

Participants with an average reduction of more than one drinking occasion during intervention weeks compared to inactive weeks were labeled responders (class 1); those who reduced by one occasion or less—or showed no reduction—were labeled non-responders (class 0). For clinical rationale and sensitivity analyses motivating the 1-drink threshold, refer to Methods, “Defining intervention effectiveness” and Supplement 5. For model performance across alternative cut-offs, refer to Supplement 7 and Table S10. We trained and tested models using single or pairwise combinations of the six candidate domains: (1) baseline alcohol use and cognitions, (2) psychological assessments, (3) sociometric nominations, (4) neural responses to alcohol and social cues, (5) peer drinking perceptions, and (6) demographics. For details on modeling specification, see Methods, “Analysis plan”, and for modeling details see Supplement 4, “Model configurations”. Full evaluation metrics—including sensitivity, specificity, PR-AUC, positive predictive value, and negative predictive value—for all feature domains are reported in Supplementary Table S7. Given the class imbalance in Study 1 (22% responders), baselines for meaningful F1 scores are 0.22 (assuming a model randomly predicts 22% positives), or 0.31 (assuming a model predicts both classes with equal probability)^44,52^.

**Fig. 1:**
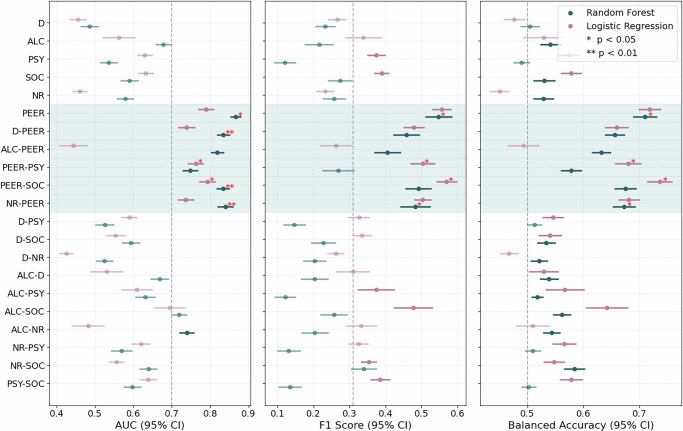
Random forest and logistic regression evaluation metrics for nested CV (k = 3, j = 5, i = 100) trained on baseline alcohol use, psychological assessments, sociometric measures, neural responses, peer drinking perceptions, demographics, and pairwise combinations, evaluated using nested cross-validation. For each model and feature domain, performance metrics—including AUC (left panel), F1 score (center), and balanced accuracy (right)—were averaged across 100 outer validation folds (30% holdout), using threefold outer and fivefold inner cross-validation (k = 3, j = 5, i = 100). Points represent mean performance, and error bars show 95% confidence intervals. Vertical dashed lines indicate the minimum thresholds for chance-level classification; due to class imbalance, the chance threshold for the F1 score is 0.31. Red asterisks indicate models that performed significantly better than chance based on permutation testing (*p* < 0.05 or *p* < 0.01); the *p* values are corrected for multiple comparisons within feature domains across the selected metrics using Benjamini–Hochberg FDR correction. Predictor domains are abbreviated as follows: D demographics, ALC baseline alcohol use, PSY psychological assessments, PEER subjective peer drinking perceptions, SOC sociometric nominations, and NR neural responses to alcohol, social cues, and at rest.

As shown in Fig. 1, results from 100 iterations of nested cross-validation across both random forest and logistic regression models showed largely comparable results, indicating that individual differences in DHI effectiveness are predictable from baseline factors (AUC = 0.79–0.87). Specifically, random forest models trained on subjective perceptions of peers’ drinking behaviors (“PEER”) achieved the highest average AUC of 0.87 (95% CI: 0.85–0.88, *p*_FDR_ = 0.020), a balanced accuracy of 0.71 (95% CI: 0.69–0.73, *p*_FDR_ = 0.020) and an F1 score of 0.55 (95% CI: 0.51–0.59, *p*_FDR_ = 0.020); exceeding the 0.31 baseline expected for equal-class predictions, given the class imbalance^44,52^. For the same domain (“PEER”), logistic regression showed a similar performance, albeit non-significant with respect to permutation tests, with an average AUC of 0.79 (95% CI: 0.77–0.81, *p*_FDR_ = 0.060), a balanced accuracy of 0.72 (95% CI: 0.70–0.74, *p*_FDR_ = 0.060), and F1 score of 0.56 (95% CI: 0.53–0.58, *p*_FDR_ = 0.060). The strongest predictor domain in the logistic regression model was the “PEER-SOC” domain, a combination of subjective peer perceptions and sociometric measures, with an AUC 0.79 (95% CI: 0.77–0.81, *p*_FDR_ = 0.010), balanced accuracy 0.74 (95% CI: 0.71–0.76, *p*_FDR _ = 0.010), and F1 score 0.57 (95% CI: 0.54–0.60, *p*_FDR_ = 0.010). Supplementary Fig. S5 presents the results for linear and radial SVMs, which also showed that the PEER domain outperformed other feature domains and achieved statistically significant predictions across all three metrics. The radial SVM achieved an AUC of 0.82 (95% CI: 0.81–0.84), F1 score of 0.552 (95% CI: 0.53–0.58), and a balanced accuracy of 0.72 (95% CI: 0.70–0.74), while the linear SVM achieved an AUC of 0.78 (95% CI: 0.75–0.81), F1 of 0.57 (95% CI: 0.54–0.59), and balanced accuracy of 0.73 (0.71–0.75).

Together, these converging results across multiple model types demonstrate that individual differences in DHI effectiveness are predictable in the given study context and highlight the robustness of the PEER domain as a key predictor of intervention response, independent of model choice.

### Identifying the strongest predictors of intervention response

To identify factors driving model performance, we analyzed feature importance in the top-performing and most parsimonious feature combination: the PEER domain. We used random forests to assess feature contributions due to their ability to capture nonlinearities and feature interactions, and since random forests allow for more direct assessment of feature importance than kernel-based SVMs^53^. The PEER domain included five self-reported features capturing participants’ subjective perceptions of their peers’ drinking attitudes and behaviors within their existing on-campus social group: (1) perceived group attitudes toward general alcohol use, (2) perceived group attitudes toward binge drinking, and (3) perceived peer approval of alcohol use. In addition, the model included two variables reflecting perceptions of high-risk peer drinking behavior: (4) perceived drinking frequency and (5) perceived drinking amount among the highest drinking peers, identified by participants as the heaviest drinkers in their social group (See Table S4 and Supplement 3, “Measures and scales”, for full measurement details).

**Fig. 2:**
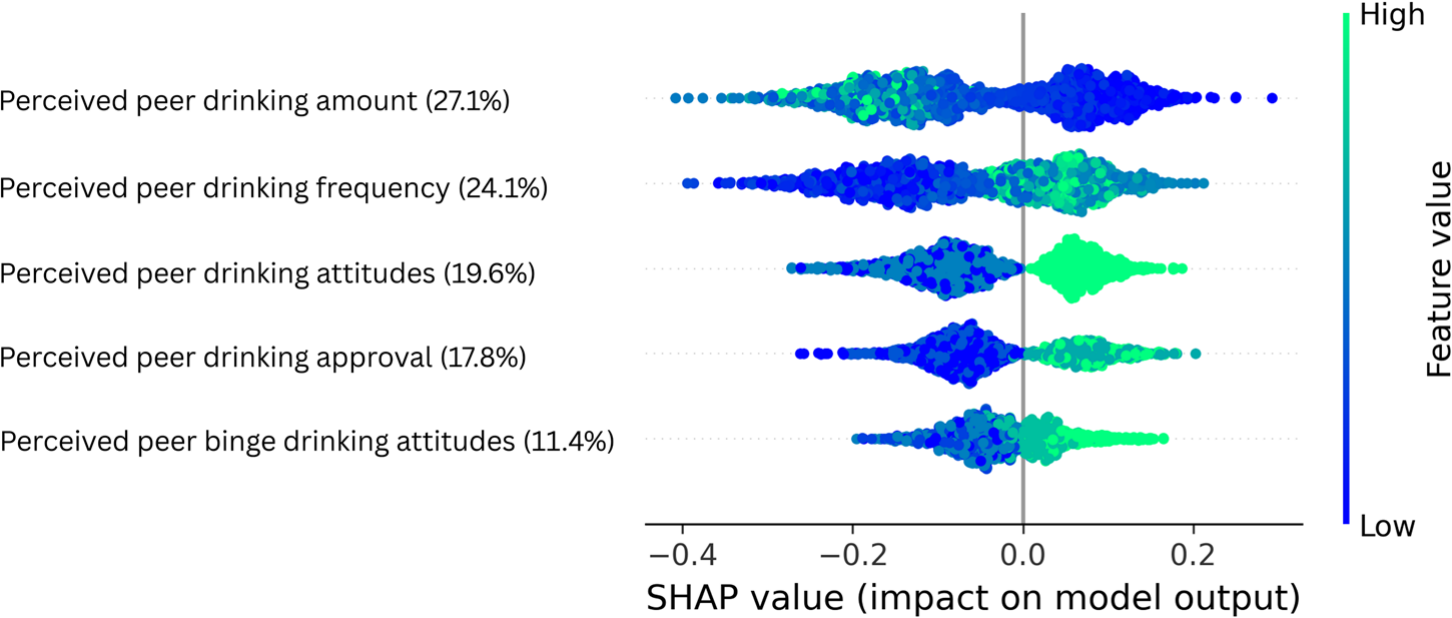
SHAP summary plot for random forest cross-validation on the best-performing feature domain (“PEER” - perceptions of peer drinking behaviors). These models included five features: perceived peer drinking amount and frequency among the nominated heaviest drinkers in one’s social group, perceived peer attitudes toward alcohol use and binge drinking, and perceived peer approval of drinking. Each row shows one feature ranked by its average contribution to the models’ predictions, with the percentage indicating the feature’s relative importance across the full model. Each point represents one prediction for a participant in one of the 100 repetitions of the nested cross-validation pipeline. The horizontal axis shows SHAP values, reflecting the impact of a given feature on the model’s output: values to the right indicate a positive contribution toward predicting intervention effectiveness (class 1), while values to the left indicate a contribution toward predicting no intervention effectiveness (class 0). Color denotes the original feature value (green = high, blue = low). This helps show how different feature values influence the direction of model predictions.

To quantify the relative contribution of each feature to model predictions, SHAP values were computed across 100 cross-validated iterations of the random forest model. These values indicate the direction and magnitude of each feature’s influence on model output, providing insights into the underlying model decisions^50^. As shown in Fig. 2, perceptions of peer drinking amount and peer drinking frequency among the heaviest-drinking peers (i.e., who were nominated as drinking the most) were the top predictors of DHI effectiveness, accounting for 27.1 and 24.1% of total feature importance, respectively. Other peer-related factors—such as perceptions of peer attitudes toward alcohol (19.6%), approval of drinking (17.8%), and attitudes toward binge drinking (11.4%)—contributed to a lesser extent. Follow-up analyses in Study 1 showed the strongest responsiveness among participants who perceived their heavy-drinking peers as consuming about 2.5 drinks per occasion and drinking on 60 to 80 occasions per year (roughly one to two times per week), a profile reflecting regular but moderate alcohol use (Supplementary Fig. S4). In contrast, perceptions of less frequent peer drinking (less than or equal to one occasion per week) were associated with a lower likelihood of predicted intervention responsiveness. Overall, these follow-up results suggest that participants who perceived the heavy-drinking peers in their social group as moderate and consistent drinkers—rather than low—were the most receptive to the psychological distancing interventions tested here.

### Predicting intervention response in an external test sample

To address RQ2—whether the best-performing models replicate in a new sample—we evaluated the PEER models trained on Study 1 data using an external test set (Study 2). Study 2 included new participants who completed a parallel psychological distancing intervention DHI protocol, allowing us to test whether the predictive patterns observed in Study 1 replicate in unseen participants. For more information on the intervention protocol of Study 2, see Methods, “Data” and “Sample”. The model, trained and optimized on Study 1, was applied without retraining to predict DHI effectiveness in Study 2. Evaluating the model from Study 1 on additional unseen data helped guard against overfitting, building our confidence that the identified relationships are not unique to the training sample. Performance metrics, including the AUC, F1 score, and balanced accuracy, were computed from a single prediction on the test set. Confidence intervals for the AUC were estimated using the analytical DeLong method, a nonparametric approach for comparing ROC curves commonly used in health studies^37,54,55^.

**Fig. 3:**
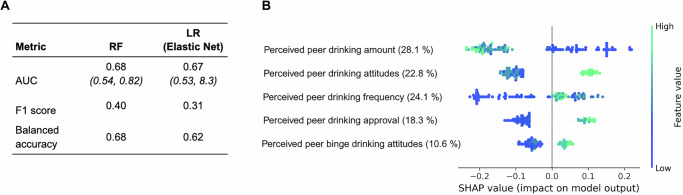
Model output summary and SHAP summary for prediction on the external test set. **A** Output summary. **B** SHAP summary. In (**A**), 95% confidence intervals were calculated for the AUC values analytically (DeLong method). In (**B**), the relative feature importance obtained from the random forest is indicated in percentage with each feature label. Each point represents the prediction on one individual’s data in the external test set. The horizontal axis shows SHAP values, reflecting the impact of a given feature on the model’s output: positive values indicate a positive contribution toward predicting effective intervention (class 1), whereas negative values indicate a contribution toward predicting no response (class 0). Color denotes the value taken on by the feature (green = high, blue = low).

On the external Study 2 test set, the random forest model achieved an AUC of 0.68 (95% CI: 0.54–0.82), an F1 score of 0.40, and a balanced accuracy of 0.68. For comparison, a random model that predicts the positive class at the base rate (12%) would achieve an F1 score of about 0.12. In turn, a model that randomly guesses each class with equal probability (50/50) would reach an F1 score of around 0.19, showing our model clearly outperformed these baselines. In practical terms, this means the random forest model correctly distinguished participants who reduced the frequency of their drinking occasions in response to the intervention (versus those who did not), with an average accuracy of 0.68. An AUC of 0.68 indicates that, for a given person who reduced their drinking and one who did not, the model will assign a higher probability of intervention effectiveness to the correct person 68% of the time. Figure 3A shows prediction results in the external test set from Study 2. The logistic regression model achieved an AUC of 0.67 (95% CI: 0.53–0.83), an F1 score of 0.31, and a balanced accuracy of 0.62, similar performance to the random forest result on the external test set prediction. The radial SVM achieved an AUC of 0.73 (95% CI: 0.59–0.87), an F1 score of 0.32, and a balanced accuracy of 0.62. The only model type that did not outperform a random baseline in terms of AUC was the linear SVM (AUC = 0.62, 95% CI: 0.48–0.77, F1 = 0.30, balanced accuracy = 0.63). The full set of evaluation metrics across all model types can be found in Supplementary Table S8.

Consistent with Study 1, perceptions of heavy-drinking peers’ behavior remained strong predictors of DHI effectiveness in Study 2. Figure 3B shows the SHAP summary and feature importance plot for the best-performing random forest PEER model on the external test set. Perceived drinking amount among the heaviest-drinking peers contributed most (28.1%), followed by overall peer alcohol attitudes (22.8%) and heavy-drinking peer frequency (24.1%). These PEER features consistently drove model decisions across both Study 1 and Study 2. This finding builds our confidence that the model captures reproducible patterns across both contexts tested here. Visualizations of feature relationships are shown in Supplementary Fig. S6.

### Follow-up sensitivity analyses

We next conducted a series of sensitivity analyses to assess the robustness of our findings across alternative modeling specifications, outcome definitions, and contextual factors related to peer perceptions and intervention specificity.

First, we tested the stability of the main random forest model performance on the PEER feature domain across different cross-validation specifications, including alternative cross-validation (k = 5) and train/test configurations (60/40 split). Our results showed consistent predictive performance (AUC = 0.83–0.85) as reported in the main manuscript (Fig. 1), indicating that predictive performance was not dependent on a particular data partitioning decision (see Supplementary Table S9). Second, we examined the impact of varying the operational definition of our main DHI effectiveness outcome—specifically the operationalization used to define intervention-driven effectiveness or behavior change. In addition to our primary operationalization of DHI effectiveness (reduction of >1 drinking occasion), we tested more lenient (reduction of >0.0 and >0.5 occasions) and more stringent (reduction of >2 occasions) thresholds. The predictive results remained consistent across alternative definitions, exceeding natural fluctuation (based on the control group, Supplementary Table S6), supporting the robustness of the findings (see Supplementary Table S10). To assess whether our main findings were driven by the psychological distancing DHIs or by natural variability in alcohol use over time (irrespective of an intervention), we examined changes in alcohol use among the control-only participants (who did not undergo the psychological distancing DHIs), with pseudo-assigned intervention sequences. In these negative control tests, model performance dropped substantially (i.e., to chance levels), suggesting that the predictive signal observed in the main analysis was intervention-specific rather than reflecting general temporal fluctuations in alcohol use (see Supplementary Table S11).

Third, we examined (a) the accuracy of participants’ perceptions of their nominated heavy-drinking peers’ behaviors compared to those peers’ self-reports, and (b) whether DHI effectiveness varied based on group drinking behaviors—that is, whether DHIs were more effective for participants embedded in social groups that drank moderately, regardless of their perceptions of peer drinking. Our follow-up analyses revealed that, on average, participants tended to underestimate their heaviest-drinking peers’ drinking in both studies (Supplementary Figs. S11, S12 and Tables S14, S15). Furthermore, we investigated the test-retest stability of peer drinking perceptions between the baseline measure and a 6-month follow-up survey, presented in Supplementary Table S17. Importantly, the heavy-drinking peers’ self-reported drinking did not predict individual differences in DHI effectiveness. In other words, an individual’s response to the intervention could not be predicted from the drinking behavior of their social group (Supplementary Table S16). These findings reinforce that participants’ subjective perceptions of their peers’ drinking behavior, rather than the peers’ actual drinking, remained the primary predictors of DHI effectiveness in the two contexts tested here.

## Discussion

Digital interventions targeting behaviors like alcohol use often show variable effectiveness across individuals^8,13^. This heterogeneity makes it challenging to evaluate their scalability or potential for widespread implementation^56^. To what extent can we predict who is likely to change their behavior, prior to intervention delivery, and which factors are most predictive of intervention effectiveness?

To address these questions, we predicted individual differences in alcohol use, following 28-day psychological distancing DHIs, across two samples of young adults who are social drinkers. We took a novel, interdisciplinary approach by evaluating multimodal predictors from different theory-driven domains—including alcohol attitudes and behaviors; psychometric assessments; sociometric centrality measures from existing peer groups; neural responses to alcohol-related and social cues; and demographics. Our findings provide two key novel insights. First, *ex-ante* prediction of individual differences in response to alcohol DHIs among young adults is feasible (train/validation balanced accuracy = 0.71; external test set balanced accuracy = 0.68), meeting previously proposed clinical-utility thresholds for identifying responders (vs. non-responders) in DHI contexts (balanced accuracy threshold = 0.67)^37,38^. Second, self-reported perceptions of peer drinking emerged as the strongest predictor of individual differences in intervention effectiveness—offering a potential theory-informed marker to adapt targeted delivery of preventive DHIs among young adults who are social drinkers. Importantly, results remained consistent across different modeling approaches and across two distinct student samples.

In both studies, we found that the psychological distancing DHIs were most effective for participants who perceived the heaviest-drinking peers within their social groups as regular but moderate drinkers—consuming approximately 2.5 drinks per occasion about 1.5 times per week (Supplementary Figs. S4, S6)—regardless of their own baseline drinking levels. In contrast, participants who perceived their heavy-drinking peers as infrequent drinkers (less than once per week) were less likely to respond to the interventions. Effectiveness further plateaued among those perceiving their peers as extremely heavy drinkers (more than three drinks per occasion). Interestingly, participants’ perceptions of how much their heaviest-drinking peers drank—rather than those peers’ actual (i.e., self-reported) drinking behavior—most strongly predicted intervention effectiveness across both studies (Fig. 1 and Supplementary Table S16). Models that included peer drinking perceptions consistently performed best (see Fig. 1 and Supplementary Fig. S5) with AUC >0.77 and balanced accuracy >0.70 on PEER features across all four model types.

With respect to peer drinking frequency, intervention effectiveness followed a consistent, nonlinear pattern: it was lowest among individuals who perceived their peers as drinking infrequently (fewer than 40 occasions per year), increased sharply for those perceiving their heaviest-drinking peers as drinking on ~40–70 occasions per year, and then plateaued for those perceiving their peers to drink above roughly 80 occasions per year. One plausible mechanism for this draws on social norms literature^57,58^: When participants perceive peer drinking approval as moderate, pro-drinking norms may be socially relevant but not extreme, allowing the psychological distancing interventions the most room to reduce the influence of social pro-drinking cues on behavior. Infrequent peer drinking and low peer approval of drinking may, in contrast, provide limited relevance to change, while extremely frequent peer drinking and strong pro-drinking approval may create a “ceiling effect”, thereby limiting intervention effectiveness. Thus, the psychological distancing interventions tested here may be most effective for individuals for whom pro-drinking norms are salient and within relevant reference points. Consistent with this account, exploratory follow-up analyses indicate that perceived group drinking approval (i.e., how much the social group approves of drinking) may partially mediate the association between perceived peer drinking and intervention effectiveness (Supplement 7 and Fig. S16), though these analyses are only intended for hypothesis generation and further validation (e.g., via experimental manipulation of perceived drinking approval) is needed to test behavior change mechanisms. Internal validation (nested CV) further showed that nonlinear models (random forest, radial SVM) performed better than linear models in terms of AUC and F1 (Supplement 6 and Table S7), suggesting that key peer drinking predictors may show nonlinear and interactive relationships with intervention responsiveness.

Our results advance the literature on DHI evaluation in several important ways. First, prior DHI evaluation studies have focused on predicting individual differences in anxiety, depression^37,38^ or eating disorder symptoms^16,17^. To our knowledge, only one study has predicted individual differences in DHI engagement and alcohol use^18^, using app log data from the first three intervention days. In contrast to these approaches^16,18,37,38^, our study predicts DHI effectiveness operationalized as behavior change, rather than intervention adherence^17,18^, with predictors solely collected at baseline. Prior DHI evaluation studies, predicting adherence and effectiveness, or their combinations, have reported AUCs between 0.616 and 0.718, with the highest relying solely on in-sample cross-validation. In comparison to a study predicting DHI effectiveness using baseline mental-health and work-related questionnaires (AUC = 0.60; balanced accuracy = 0.60)^16^, our model demonstrated stronger performance, with an AUC of 0.87 (random forest balanced accuracy = 0.71, 95% CI: 0.689–0.732; logistic regression balanced accuracy = 0.72, 95% CI: 0.698–0.739) on the internal test set. Critically, our model maintained performance on an external dataset (AUC = 0.68; balanced accuracy = 0.68; meaning it correctly classified responders and non‑responders 68% of the time). The improved performance of our model may, in part, reflect theory-driven feature selection, i.e., the inclusion of peer drinking perceptions (see Supplement 1 for theoretical motivation), which narrows the predictor space and avoids excessive multidimensionality. In contrast to prior work on digital mental-health interventions, where baseline psychological assessments (e.g., depression and anxiety symptom measures) were the strongest predictors of symptom reduction^16^, similar measures in our study (“PSY” domain) showed limited predictive power. These findings likely reflect contextual differences in intervention targets: while psychological questionnaires are central in digital mental-health contexts, the perceived social context is possibly more relevant for predicting changes in alcohol use among young adult social drinkers.

Results should be interpreted in light of the study’s strengths and limitations. Key strengths include the integration of theory-driven feature sets spanning psychological, social, and neural domains, collected prior to intervention delivery, to predict behavior change, with successful replication across two samples and across a wide range of modeling specifications. Our results advance prior DHI literature, where in-app engagement metrics are often used in predicting outcomes, but are limited as predictors because interaction with DHI content does not necessarily indicate meaningful behavior change^17,34,35^. ML models built on weakly theory-grounded, or narrow predictor sets (e.g., demographics alone) risk overlooking critical psychological, behavioral, and contextual factors that can influence intervention response^59,60^. Furthermore, the lack of external out-of-sample validation in many studies raises concerns about reproducibility and generalizability across populations, settings, and intervention types^33,61^. Our results show that intervention effectiveness can be predicted out-of-sample from baseline peer drinking perceptions across four model types: two nonlinear models (radial SVM, random forest) and two linear models (linear SVM, logistic regression). All models, including PEER features, exceeded baseline thresholds in internal cross-validation. External test set prediction remained significantly above chance-level in all four model types except linear SVM, where the 95% CI for AUC crossed the 0.5 threshold (0.62, 95% CI: 0.48–0.77), overall indicating that the models captured meaningful patterns. Model-free analyses (Supplementary Table S18) further showthat all PEER variables differ significantly between intervention responders vs. non-responders (*p* < 0.05), increasing our confidence that the PEER feature domain contains a meaningful predictive signal independent of model complexity.

A key consideration is the level of accuracy that is clinically meaningful when evaluating DHI effectiveness. Although no universal benchmark exists, prior work suggests that even moderate accuracy^62^ can effectively guide intervention decisions. For example, a trial of internet-delivered CBT for insomnia indicated that a predictive model identifying individuals at risk of treatment failure with ≥67% balanced accuracy could guide adaptive DHI allocation, leading to improved health outcomes^37,38^. In our study, models achieved a balanced accuracy of 0.71 in cross-validation and 0.68 on the external test set, successfully meeting this benchmark. Thus, our predictions of intervention responsiveness achieve a level comparable to that of models used to guide adaptive clinical decisions in the digital health literature.

With respect to limitations, Study 1 took place while participants were present on university campuses and took part in an in-person study visit that included instructions for the psychological distancing intervention (see refs. ^12,43^). Study 2, our external test set, was conducted during the COVID-19 pandemic, introducing contextual differences from Study 1. While Study 1 involved participants largely on campus, Study 2 was conducted entirely remotely (see refs. ^12,43^ for recruitment details); with lower overall drinking levels (Study 1: 3.08 drinks/occasion on inactive weeks vs. 2.86 drinks/occasion on active weeks, 2.20 occasions/week on inactive weeks vs. 1.83 occasions/week on active weeks; Study 2: 2.06 drinks/occasion on inactive weeks vs. 2.23 drinks/occasion on active weeks, 2.08 occasions/week on inactive weeks vs. 1.80 occasions/week on active weeks; Supplement 7 and Table S12), lower adherence (median prompt completion 90.3 in Study 1 vs. 82.1% in Study 2; Supplement 2 and Table S1), and lower intervention responsiveness (22% in Study 1 vs. 12% in Study 2; Supplement 2 and Table S1). The lower response rates in Study 2 may possibly reflect contextual differences related to the COVID-19 pandemic, including changes in social environments such as reduced in-person contact with peers. Participants in Study 2 were slightly more likely to report drinking with family rather than with peers: Study 1 participants reported drinking with peers on 61% of occasions, compared to 15% of occasions with family, 10% with a significant other, 6% with strangers, 5% alone, and 3% with coworkers. In Study 2, which coincided with the COVID-19 pandemic, participants drank with peers 48% of the time, with family 23%, with a significant other 12%, with strangers 3%, alone 13%, and with coworkers 1% of the time. Together, these differences introduce potential confounds related to drinking behavior and peer interactions. Despite these differences, intervention adherence among responsive vs. non-responsive participants remained overall high (> 80% of prompts completed) across both studies. Importantly, Study 2 was not used for model training or internal validation and had no participant overlap with Study 1, serving as a fully external test set that mirrors real-world deployment; i.e., where models trained in one setting may be applied to make predictions in new temporal settings. Although the shift in contextual drinking environments between Study 1 and Study 2 presented pandemic-related confounds, our predictive model nonetheless maintained balanced-accuracy performance above the 67% clinical-utility benchmark proposed in prior work^37,38^. Notably, despite these confounds, we view the availability of an external dataset as a methodological advantage to test model robustness. Among prior DHI evaluation studies (refs. ^16–18,37,38^), only refs. ^16,37^ evaluate model predictions using a held-out test set, while other studies rely on repeated within-sample cross-validation alone.

Interestingly, on average, participants underestimated the drinking levels of their heaviest-drinking peers in both studies, and to a similar degree. That is, peer nominations were not more inaccurate when students were not physically together (Study 2) vs. on campus (Study 1) (Supplement 7, Figs. S11, S12, and Table S14). The observed underestimation of peer drinking may partly reflect more general limitations of quantity–frequency drinking measures, which can miss heavy-episodic or irregular drinking in college-aged samples^63,64^, or the nature of the student sample from two selective colleges^65^. Future research may incorporate diary-based methods to capture more granular event-level variability in peer drinking (e.g., heavy binge drinking episodes) and examine patterns of peer drinking estimates beyond highly competitive college student populations. Perceived peer drinking frequency showed moderate test-retest reliability for drinking frequency (*r* = 0.47, *p* = 0.007) but lower stability for perceived drinking amount (r = 0.20, *p* = 0.279) from baseline to 6-month follow-up in Study 1 (Supplement 7 and Table S17). The lower stability in perceived peer drinks per occasion may, in part, reflect peer turnover in participants’ high-drinking peer groups, as only one in four of the nominated heavy-drinking peers (on average) remained the same across the 6 months (Supplement 7 and Table S17). Another limitation is that we did not collect data on grams of ethanol consumed per occasion, which prevented us from applying clinical endpoints for alcohol reduction^66^. Our >1-occasion/week DHI effectiveness threshold represents a measure of change within the context of the study design and based on preventive literature on alcohol risks^67–69^. This threshold exceeds normal week-to-week fluctuations in drinking occasions that we would expect in the absence of an intervention, i.e., among control participants (Supplementary Fig. S3 and Table S6), and aligns with prior evidence that even modest reductions in drinking frequency are associated with lower risk of alcohol-related harms, cardiovascular disease, and all-cause mortality^67–69^. While smaller reductions in drinking may still confer health benefits—for example, prior studies have considered frequency decreases as small as 0.3–0.4 days per week meaningful behavior change^70,71^, particularly when sustained over time and across larger samples—the >1-occasion/week threshold aims to ensure that the observed intervention response is less likely to reflect random variability. From a prevention standpoint, future research could identify the minimum magnitude of weekly alcohol‑use reduction (measured via ethanol intake) linked to measurable benefits to health, well-being, productivity, and long-term risk of disease among individuals without alcohol use disorders. Sensitivity analyses further confirmed that the observed effects were specific to the psychological distancing DHIs and not attributable to natural variability in alcohol use over time (Supplementary Table S11), strengthening our confidence in the robustness of the findings. Finally, due to sample size limitations, we were unable to isolate the effects of the different psychological distancing strategies. Future studies with larger samples can isolate psychological distancing mechanisms and further clarify sources of heterogeneity in intervention response.

Another important limitation is the relatively small sample size and notable class imbalance in our data. To mitigate these concerns, we followed established guidance ML with small sample sizes and imbalanced classes^72^. To avoid risk of overfitting, models were trained separately on six predefined feature domains (up to 20 features per domain; maximum of 36 features for pairwise combinations; Supplementary Table S2), with the best-performing model using only five PEER (peer drinking perception) features. Model complexity was constrained by limiting the number of estimators, restricting tree depth, setting minimum thresholds for node splits and leaf sizes. All models were trained using nested cross-validation, and validation was assessed on a strictly held-out, external test set (Study 2). We report and interpret metrics appropriate for imbalanced classes (balanced accuracy and F1 score) and report model performance using additional metrics for transparency (refer to Method, Analysis plan for details). Model stability was evaluated using multiple performance metrics with confidence intervals from 100 nested CV runs. Permutation tests provided empirical null distributions, which indicate that the observed model performances significantly exceeded chance levels and that the predictive signal was robust rather than driven by small-sample variability. Although the predictive models showed promising out-of-sample performance, and we took robust methodological safeguards, replication in larger and more diverse populations is needed to test the robustness of the findings.

Both Study 1 and Study 2 samples consisted of distinct, non-overlapping college students who are social drinkers from two urban Northeastern universities considered very selective^65^. Participants self-identified as predominantly white (55.2% in Study 1 and 38.6% in Study 2), which limits the generalizability of the findings to other racial or ethnic groups, students at different types of colleges, non-college young adults, and individuals with alcohol use disorders. Participants also predominantly identified as women (61% in Study 1and 80% in Study 2), and no participants identified as non-binary. This gender imbalance may in part reflect the broader SHINE project recruitment strategy, where in both studies, participants were recruited through on-campus social groups in which 80% of more group members expressed interest in participating and a total of eight groups were women-only (See Methods, “Sample” for group-based descriptives). The observed gender imbalance may also be, in part, associated with broader trends in college-aged research participation, where women, in general, may be more likely to enroll in health and psychology studies^73^. This imbalance has implications for alcohol-related research, as college-aged men, on average, are expected to consume more alcohol and experience more alcohol-related harms than women^74^, though these gaps are increasingly narrowing^74^. Further, individuals who identify as non-binary may face distinct stressors that contribute to patterns of alcohol use and intervention response^75^. Participants from different racial and ethnic backgrounds may also show varying baseline drinking levels and intervention responsiveness. We observed higher participation of Black/African American participants in Study 2 vs. in Study 1; however, the small subgroup sizes in the current study limited stratified analyses by race/ethnicity. Future research should adopt recruitment strategies that yield more demographically diverse samples, including balanced gender distribution and broader racial/ethnic diversity. Larger and more heterogeneous samples are needed to assess whether, and to what degree, model performance and intervention effectiveness differ across gender and racial/ethnic groups; to better understand heterogeneity in DHI responsiveness. In addition, the neural measures we examined here are limited in scope. Previous research indicates that multivariate^76^ and network controllability^12,28,77,78^ metrics are associated with intervention response in the current sample. Thus, additional neural features not included in our study may potentially capture additional variability in DHI response. Furthermore, while SHAP values offer insights into model-driven feature importance, they only reflect the associations that the model has learned. SHAP values should not be interpreted as evidence of causality in a behavioral sense. That is, they do not imply that changing a given feature would directly alter an individual’s response to the intervention. However, SHAP still serves as a powerful tool for hypothesis generation, helping to identify potentially influential intervention response factors for further experimental investigation.

Despite these limitations, the study carries important practical considerations regarding adaptive DHI delivery. In practice, *ex-ante* screening for DHI responsiveness may be implemented with a brief, low-cost questionnaire using peer drinking perception items, the strongest predictors of intervention response in our data. Based on initial PEER survey responses, individuals could be provisionally classified as likely responders or non-responders. Consistent with prior work^16^, predicted non-responders could enter a short monitoring phase involving brief ecological momentary assessments or coarse geolocation data to track drinking behaviors and social drinking context. Depending on observed patterns, non-responders could then be offered context-sensitive alternatives such as targeted smartphone-based psychological distancing prompts combined with optional normative-feedback content^79^, additional prompts on alcohol-related harms, or short virtual check-ins with interventionists. Predicted responders, by contrast, could proceed directly with the standard psychological distancing DHI protocol. Contextual drinking patterns in our samples further suggest opportunities for such targeted adaptations. Our data shows that participants drank primarily in social situations—most often with peers (61% in Study 1; 48% in Study 2)—indicating that a small set of recurring social contexts accounted for much of their alcohol use. Drawing from the growing just-in-time adaptive intervention literature^80^, integrating more granular contextual assessments (e.g., drinking at a bar, restaurant, house party, Greek party, vs a small gathering with close friends) could support more precise, event-based prompt delivery among potential non-responders. For example, psychological-distancing reminders could be delivered at moments or at locations where individuals are most likely to drink with peers, rather than the fixed twice-daily prompt delivery. Aligning DHI content with individuals’ real-world drinking environments may improve the relevance of support and thereby increase the potential for intervention response, especially among individuals initially flagged as non-responders.

Cost-benefit considerations also favor the piloting of this adaptive approach as misclassification carries minimal risk in the preventive, non-clinical context of this study. Positive predictive value—the probability that individuals predicted to respond actually do respond—was relatively low (0.51), meaning roughly half of predicted responders ultimately respond. While this level of accuracy would be limiting in higher-stakes clinical settings, it may be acceptable here because all participants still receive a non-harmful intervention. False negatives (predicted non-responders who might have responded) still receive the standard DHI, and false positives simply receive a slightly more intensive but still low-cost variant. Taken together, this account illustrates how context-sensitive DHI tailoring, informed by ML models that predict individual differences in intervention response, could be implemented in practice. However, replication of these predictive models in larger, more diverse samples, along with prospective evaluation of whether algorithmic adaptations meaningfully improve intervention effectiveness and optimize resource allocation, is needed. Future research may test the adaptive pathways proposed here, e.g., the targeting of context-sensitive psychological distancing prompts, and assess the extent to which ML-guided, context-sensitive DHI tailoring is feasible, scalable, and cost-effective. Finally, it is important to acknowledge that the baseline for clinical usefulness (67% balanced accuracy) used as a heuristic was based on empirical findings from prior studies in DHIs delivering cognitive behavioral therapy to treat insomnia, depression, anxiety, and panic disorder^37,38^, which may not directly generalize to preventive alcohol use interventions targeting healthy individuals. Future studies are needed to establish empirically grounded baselines for acceptable accuracy thresholds in stratifying participants for different routes of preventive DHIs, to better determine when predictive models provide meaningful health outcomes and cost benefit over one-size-fits-all solutions. Understanding these patterns is critical for maintaining effectiveness and scalability as DHIs move from controlled studies to diverse, real-world populations^33^. In response, the DHI evaluation framework proposed here directly aligns with global calls to leverage digital health, artificial intelligence, and data-driven interventions to improve the accessibility, quality, and equity of healthcare (core aims of the UN’s Sustainable Development Goal 3: Good Health and Well-Being^81^).

Overall, this study introduces a novel, multimodal approach for evaluating individual differences in the effectiveness of DHIs and demonstrates the feasibility of using ML to predict responses to a preventive alcohol DHI among young adults. In the context of psychological distancing DHIs, our findings show that individual responsiveness can be feasibly predicted before intervention delivery. Our models achieved moderate predictive performance and replicated across two college student samples across varying temporal contexts. By integrating behavioral, psychological, social, and neural data, we identified peer drinking perceptions as the most robust, parsimonious, and scalable predictor of intervention response, highlighting the value of context-aware, theory-informed modeling. Interventions were most effective for participants who perceived their peers as moderate but frequent drinkers. Findings support the feasibility of identifying likely intervention responders using a cheap, low-burden screening tool (peer drinking perceptions) in a preventive social drinking context. This work provides a framework to measure treatment effect heterogeneity in DHIs. Future work may apply this framework for more targeted, scalable, and equitable delivery of digital interventions.

## Methods

### Ethics approval

All research, methods, and experimental procedures were conducted in accordance with, and were approved by, the Institutional Review Board (IRB) at the University of Pennsylvania and acknowledged by the Human Research Protection Office of the Department of Defense. All participants provided informed consent prior to participating in the study and received financial compensation. For more details on compensation, refer to Ref. ^43^.

### Data

We used data collected as part of the Social Health Impact of Network Effects (SHINE) study^43^, designed to investigate factors that influence well-being and health-related behaviors in young adults. College students, part of on-campus student groups, were recruited from two private, selective urban universities in the Northeastern United States^65^. Participants took part in one or more components of the study, including surveys, social network assessments, functional and structural neuroimaging scans, and a 28-day alcohol use intervention paired with ecological momentary assessment (EMA), administered twice (referred to as Study 1 and Study 2)^12^. For details on participant eligibility, recruitment, and study procedures, see ref. ^43^. Supplementary Fig. S1 illustrates the data collection modalities relevant to the current investigation. For additional studies using components of this data beyond the focus of the current investigation, see refs. ^12,28,77,78^. Data collection took place between January 2019 and April 2021. Study 1 was collected prior to the COVID-19 pandemic, whereas Study 2 coincided with the COVID-19 pandemic.

### Study procedures

After providing informed consent, participants completed an online baseline assessment including demographic information, self-reported alcohol-related attitudes, peer perceptions, and behaviors, as well as psychological assessments. As part of the baseline assessment, participants completed a sociometric nomination survey to identify peers within their social group across dimensions such as alcohol use and social influence. Following the baseline assessment, participants were randomized into two psychological distancing intervention conditions and a control condition. Those assigned to the intervention received a brief psychological distancing training instructing them to mentally distance themselves from alcohol cues by focusing on the present moment or adopting the perspective of low-drinking peers^12^. Training details can be found in Supplement B of Ref. ^12^, and instruction material is publicly available^82^. A subset of participants completed functional MRI scans, including an alcohol cue reactivity task and a resting-state scan. Additionally, participants were invited to participate in a 28-day smartphone-delivered alcohol use intervention, paired with an EMA protocol, described below. The current study represents a retrospective analysis of data collected during the SHINE EMA intervention component.

### Sample

The Study 1 cohort comprised the training and internal validation set, and the Study 2 cohort comprised the external test set. In Study 1, 612 participants completed a baseline survey assessment that included demographic information, self-reported alcohol use, psychological assessments, social group and peer perceptions, and sociometric nominations. A subset of 113 participants underwent functional MRI scanning. Out of these, 72 participants were assigned to the psychological distancing interventions. Additionally, 41 participants were assigned to the control condition of the 28-day intervention, which were excluded due to not receiving “on” and “off” intervention weeks. Of the 72 participants assigned to the intervention, 69 participants completed the EMA protocol. Of these, an additional two participants were excluded for having more than 20% missing features across all feature domains; a threshold chosen based on best practices to balance data retention and quality^83^. The final training and cross-validation sample, therefore, consisted of 67 participants, who provided multimodal data spanning behavioral, psychological, social, and neural domains (see Supplementary Fig. S1).

In Study 2, a total of 377 participants completed the baseline survey, which collected information on demographics, peer drinking perceptions, and sociometric measures. Of the 279 participants randomized to the EMA protocol, 185 were assigned to the psychological distancing interventions, while 94 participants were excluded for being in the control condition. Among the 185 intervention-assigned participants, *n* = 33 had previously participated in Study 1, and were thus deemed ineligible. From the 152 eligible participants, *n* = 5 were excluded or dropped out before data collection (no data), and *n* = 33 were excluded due to missing data (equal to or more than 20% of features missing). This resulted in a final test set of 114 participants. The final Study 1 analytical sample included 93% of all eligible participants who underwent the psychological distancing interventions, while Study 2 included 78% of all eligible, non-overlapping participants. Refer to the participant flowchart in Supplementary Fig. S1 for details on participant exclusion. In both studies, participants assigned to the intervention conditions and the control group were comparable on key demographic variables (e.g., age, gender, race/ethnicity) and baseline drinking amount measures (see Supplementary Table S3). Study 1 control participants reported higher drinking frequency compared with participants in the intervention conditions; however, this group difference was not observed in Study 2. Overall, we found largely no systematic differences between intervention and control groups. Participants receiving the mindfulness and participants receiving the perspective-taking strategy were pooled together in all analyses reported in the main manuscript in line with our prior report on the main intervention effects^12^ and given sample size considerations.

Participants were recruited from a variety of campus groups, including performing arts groups (40% in Study 1; 36% in Study 2), sororities/fraternities (20% in Study 1; 32% in Study 2), sports clubs (30% in Study 1; 23% in Study 2), technology clubs (0% in Study 1; 5% in Study 2), and other student organizations (10% in Study 1; 5% in Study 2). Among these, two groups in Study 1 (20%) and six groups in Study 2 (27%) were women-only organizations—primarily sororities and performing arts groups.

### Sample size considerations

The target sample size for Study 1 was based on a power analysis conducted for an MRI component in the original grant (refer to protocol ref. ^43^). However, recruitment was interrupted early due to the COVID-19 pandemic, resulting in *n* = 113 participants who were randomized to psychological distancing conditions or the control condition. From this sample, *n* = 72 were assigned to an intervention, *n* = 69 completed the EMA protocol, and *n* = 67 had complete data (≤20% of features missing) and were thus included in the final analyses. Study 2 was open to all on-campus social groups who expressed interest in participating in Study 1, with no formal power analyses conducted.

There is no universally accepted minimum sample size for meaningful prediction with random forests or logistic regression, other than rules of thumb which are highly contested^84,85^. The minimum required number of samples depends on the use case and underlying effect sizes. However, there is precedent for using random forest classification with similar sample sizes in biomedical research^86^ and we took rigorous steps to avoid overfitting, following established guidance for ML with small sample sizes^72^ (see Methods - Analysis plan).

### Psychological distancing interventions

For detailed information on the DHI design and ecological momentary assessment protocol, see ref. ^43^. Participants in the psychological distancing interventions received smartphone-delivered prompts during “on” weeks, alternating with “off” (inactive) weeks in a within-person crossover design. Each day, participants received four prompts: two intervention reminders at 2:00 pm and 9:00 pm, encouraging psychological distancing strategies (mindfulness or perspective-taking), and two alcohol use surveys at 8:00 a.m. and 6:00 p.m. For intervention prompts, see ref. ^82^. Intervention conditions alternated weekly in a counterbalanced within-person ABAB or BABA design, with participants blinded to week order. Alcohol use was assessed with a single item asking whether they had consumed alcohol since the previous survey: “Since your EVENING/MORNING survey, have you consumed any alcohol? (“No” or “Yes” response option). This intervention protocol was implemented twice—once in Study 1 and once again in Study 2—with parallel procedures across both studies. Given our primary focus on modeling individual differences in DHI effectiveness, the main analyses include only participants assigned to the intervention, i.e., psychological distancing conditions. Sensitivity analyses incorporating control participants are reported in Supplementary Table S11.

### Measures

We characterized participants across behavioral, psychological, social, and neural domains using multimodal data across six domains: baseline alcohol use and perceptions, psychometric assessments, subjective social group perceptions, sociometric nominations, demographics, and neural responses to alcohol cues, social cues, and at rest, resulting in a total of 62 candidate predictor features. For the full list of measure descriptions and scales across all modalities, see Supplementary Table S4. For all measures collected within the SHINE project, beyond the scope of the current study, refer to the SHINE protocol^43^.

### Defining intervention effectiveness

Our main outcome is a binary indicator of intervention effectiveness, evaluated through a prognostic classification task. Participants were classified as having successfully changed their behavior in response to the intervention if they demonstrated a reduction in drinking frequency by an average of more than one drinking occasion between intervention weeks (on-weeks) and inactive weeks (off-weeks). This threshold was chosen to align with the intervention’s main effect (reducing the frequency of alcohol consumption^12^) and to reflect an interpretable and meaningful effect size. Specifically, a reduction of >1 occasion per week was defined a priori as the smallest interpretable change given our study’s measurement resolution. Because the trial alternated two intervention and two inactive weeks, the minimum detectable difference between conditions is an average reduction of 0.5 drinking occasions per week. In the control group (who did not undergo active weeks), week-to-week fluctuations averaged −0.18 (SD = 1.21), meaning that random variation can reach roughly ±1.4 occasions (see Supplementary Fig. S3 and Supplementary Table S6). Setting the threshold at >1.0 per week, therefore, ensures that only reductions exceeding normal week-to-week noise (i.e., equal to or above −1.5 occasions over two weeks) are classified as meaningful behavior change. In prior studies, frequency reductions as small as 0.3–0.4 days per week have been considered meaningful in brief alcohol interventions, given their low intensity and large population reach^70,71^. Moreover, independent of total consumption, drinking frequency itself predicts health risk: having alcohol-free days is associated with lower cardiovascular and all-cause mortality than daily drinking^67^, and drinking frequency independently influences cardiovascular risk beyond the average amount consumed, and even small changes in weekly intake are associated with measurable differences in health outcomes^68,69^. Our >1-occasion cutoff therefore represents a conservative, interpretable reduction that exceeds random variation and aligns with frequency-based improvements in health outcomes. Moreover, sensitivity analyses were conducted using lower and higher intervention effectiveness thresholds (reduction by >0.0, >0.5, or >2 occasions per week, respectively). We observed consistent results across different operationalizations of intervention effectiveness, exceeding natural fluctuation.

### Data preparation

**Fig. 4:**
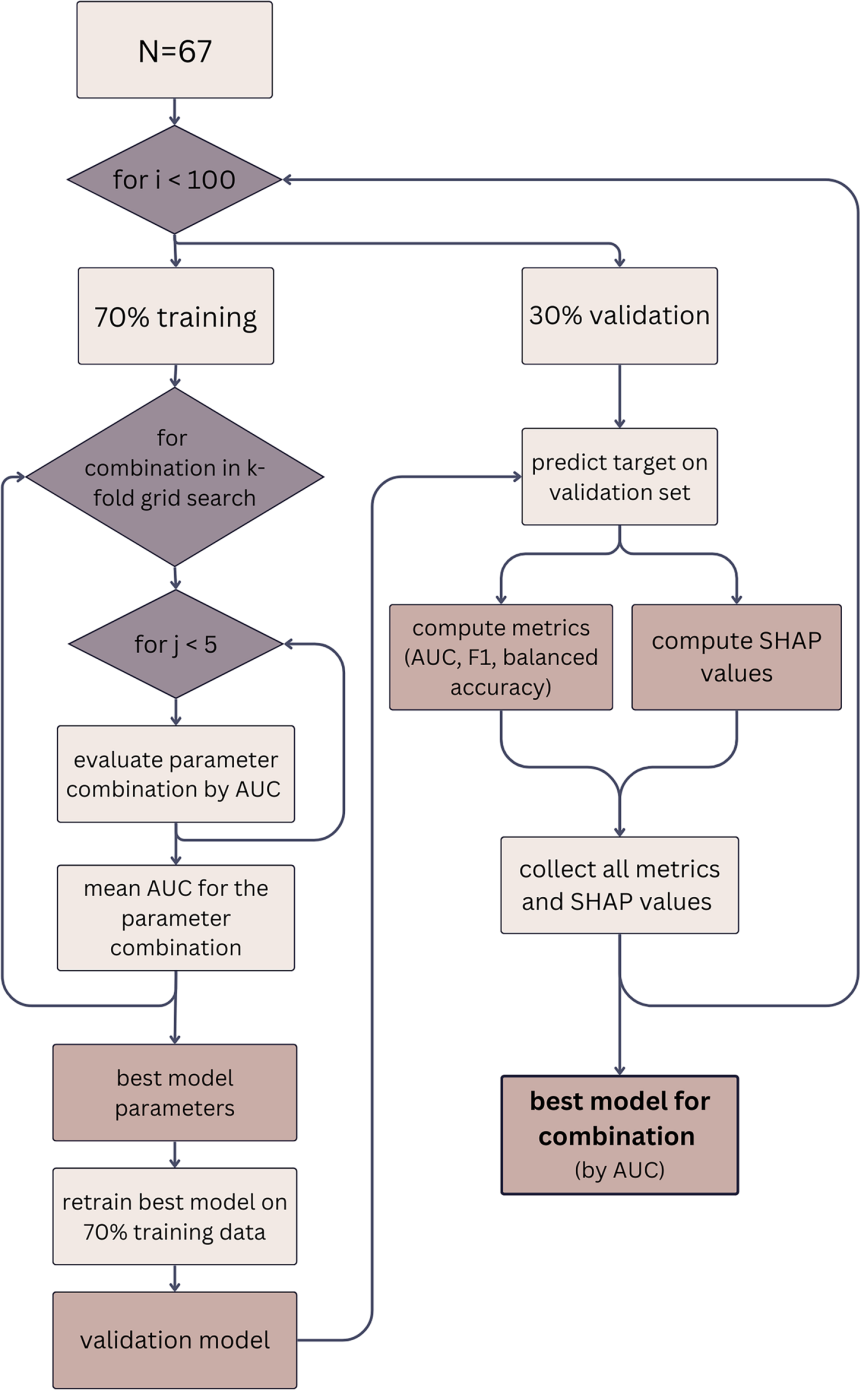
Training and validation workflow for evaluating models on all feature domains in Study 1. The process was repeated for each of the six feature domains and pairwise combinations of domains.

To reduce multicollinearity between features, a correlation analysis was first conducted within each of the six feature domains using the Study 1 dataset. Variable pairs with Pearson correlation coefficients >0.8 were removed^87^. This process was applied to six out of the total 62 features (three from psychological assessments, two from sociometric nominations, and one from the neural responses to alcohol and social cues domain). Following this step, a total of 56 features remained for prediction. See Supplementary Table S4 for all variables included in modeling. Missing values were imputed using median imputation separately within each data split to avoid data leakage, following best practices^88^. Specifically, imputation was performed independently for the training and validation splits during each fold of the inner cross-validation loop (see Fig. 4), using only data available within that split. This procedure affected two features: income (7.5% missingness) and the social anxiety and inhibition scale (4.5% missingness).

Change in alcohol use frequency was calculated for each participant by computing the mean number of drinking occasions per week during intervention (on) weeks and during inactive (off) weeks. Participants were classified as responders (target class 1) if the mean during intervention weeks was more than one occasion lower than during inactive weeks (i.e., difference >1.0) and as non-responders (target class 0) otherwise.

### Model development

To examine RQ1—whether psychological, social, behavioral, neural, and demographic data can predict intervention effectiveness (i.e., reductions in alcohol use frequency)—we used supervised ML, and specifically a random forest classifier, an ensemble learning method that combines the predictions of many decision trees^53^, as our primary model. For the full model configuration, refer to Supplement 4. Random forest was selected based on priors from related digital health studies, which employ tree-based classifiers to predict DHI outcomes (intervention completion/goal achievement and eating disorder symptom improvement, respectively)^17,18^, and has been adopted in related small sample contexts^86^. Random forests were also selected for their ability to handle high-dimensional data, a particular challenge with this dataset, as well as nonlinear feature-outcome relationships and feature interactions. Given the importance of interpretability in health-related ML tasks, random forests typically provide a balance between predictive performance and model transparency, enabling feature importance analysis through SHAP values^50^. In line with prior work, which often compares multiple models on the same prediction problem for transparency^16,18,54^, we provide a simpler logistic regression model (penalized with Elastic Net) as a baseline comparison. Logistic regression offers a linear approach and is typically less robust in cases with high-dimensional data^85^, however, it is often used in clinical and behavioral contexts^16,89^, especially when sample sizes are small. Furthermore, we assess two SVM models (linear and radial), which are also commonly used in intervention outcome prediction tasks^16,54^. Thus, we provide a comparison of four model types: Two more complex, nonlinear models (radial SVM and random forest), and two simple linear models (linear SVM and logistic regression). This model comparison helps build our confidence that predictive performance captures true feature-outcome relationships vs. spurious artifacts of model choice or high model complexity. For simplicity, random forest and logistic regression effects are presented in the main manuscript, and results from the two SVM models are presented in the supplements (Supplement 6, Table S7, and Fig. S5).

We evaluated the predictive performance of models trained on six theory-driven feature domains: alcohol use and cognitions, psychological assessments, sociometric nominations, neural responses to alcohol cues, social cues and at rest, peer drinking perceptions, and demographics. We trained separate models on each unimodal domain (six models) and on all possible pairwise combinations of these modalities (15 two-way combinations), resulting in a total of 21 feature combinations. This exhaustive pairwise approach allowed us to identify complementary feature sets while mitigating the risk of overfitting and inflated multiple comparisons in small samples. Importantly, no single model was trained using all 56 features. As shown in Supplementary Table S4, the maximum number of features within a single domain was 20, and the maximum number of features across any combination of two domains was 36. The best-performing model reported in the manuscript, trained exclusively on the PEER feature category, utilized only five features.

To ensure robust model evaluation and prevent information leakage, a nested cross-validation framework was employed on the training data from Study 1 (*N* = 67) with an outer loop for model validation and an inner loop for hyperparameter tuning across a predefined parameter grid, which can be found in the Supplementary Table S5. To prevent overfitting, we followed accepted guidance for ML in small sample sizes^72^. Key hyperparameters were restricted by limiting the number of estimators, constraining tree depth, setting minimum thresholds for node splits and leaf sizes for random forests, and by applying an Elastic Net penalty in logistic regression (see model parameters in Supplementary Table S5). Nested cross-validation was performed, and an external test set was left completely out of the training and cross-validation procedure. In each outer-loop iteration (100 repetitions), the data were randomly split into a 70% training set and a 30% validation set, stratified by the outcome variable to ensure that the base class distribution was representative of the full sample. Within each training set, nested loops performed hyperparameter selection using threefold cross-validation, with each fold tested five times per parameter combination. The best-performing hyperparameter set, determined by averaging results across inner-loop repetitions, was then used to train the validation model on the full 70% training data, which was subsequently evaluated on the 30% validation set. After the 100 repetitions, a final best model for the feature combination was found based on its AUC performance on the validation set. To evaluate model performance, we report multiple, complementary metrics: AUC, commonly reported in classification problems in healthcare^90^, assesses the model’s ability to correctly rank positive and negative cases based on predicted probabilities, but does not reflect how well the model identifies a meaningful decision boundary. To address this limitation, we also report balanced accuracy and F1 score to directly evaluate classification performance. Balanced accuracy was preferred over standard accuracy as it accounts for both sensitivity and specificity, ensuring equal weight is given to positive and negative predictions^44^, a crucial characteristic given the class imbalance in both the training/validation set (Study 1) and the external test set (Study 2) containing 22.7% and 12.1% positives, respectively. The F1 score, while optimizing the balance between precision and recall, primarily focuses on the correct classification of positives^44^. Since correctly identifying both classes is equally important, balanced accuracy was reported alongside the F1 score to provide a robust and fair assessment of model performance. Additional metrics—sensitivity, specificity, precision-recall AUC, positive predictive value (PPV), and negative predictive value (NPV)—were reported for transparency (see Supplementary Table S7). Validation metrics were reported as an average over all validation folds. Confidence intervals were estimated for each evaluation metric across the 100 obtained values due to the outer loop repetition. Prediction success was defined as statistically significant above-chance performance (*p* < 0.05) in internal cross-validation, using permutation testing. We applied SHAP to quantify the contribution of individual features to each model’s predictions, improving model transparency and interpretability^50^.

This nested cross-validation approach was used to provide an unbiased estimate of model performance by ensuring that hyperparameter selection occurred independently from the final evaluation and closely follows that employed in ref. ^17^. Figure 4 illustrates the full workflow.

To assess whether model performance exceeded chance levels, we conducted permutation tests for each feature domain by randomly shuffling the outcome labels in the training set (Study 1) while preserving the original feature structure. For each of the 100 iterations, we ran the full training and evaluation pipeline—including nested cross-validation and hyperparameter tuning—on the permuted data to generate a null distribution of evaluation metrics (i.e., AUC, F1, and balanced accuracy scores). We then calculated the one-sided *p* value for each metric as the proportion of the permutation-based metric that was equal to or greater than the mean metric observed in the original (unshuffled) model. This procedure tests whether the model’s observed performance is statistically distinguishable from random classification, ensuring that it captures a genuine association between predictors and the outcome rather than chance patterns^91^. Within each domain, we then controlled for multiple testing across metrics using the Benjamini–Hochberg false discovery rate procedure at *q* = 0.05^92^. This approach controls for inflated false positives due to evaluating multiple metrics while maintaining focus on domain-level performance.

### Model evaluation

To examine RQ2—whether the best-performing model from Study 1 replicates in a new sample of unseen participants—we evaluated the best performing model on a non-overlapping, external test sample from Study 2 (*N* = 114) without any additional model tuning. None of the individuals included in Study 1 were part of the Study 2 sample. Following established practice^72^, we report all performance metrics for prediction on the external test sample. The variance of the AUC is calculated using the DeLong method^37,54^, providing a 95% confidence interval for this metric. Because participants in Study 2 were completely non-overlapping with participants in Study 1, this evaluation tests the model’s external validity—that is, how well it generalizes to new data. A model that had simply memorized patterns from the training data (i.e., was overfitted) would perform poorly on this unseen test set. Consistently strong performance (i.e., performance significantly exceeding that of baselines set through permutation tests and a small/moderate drop in accuracy compared to training/validation) across studies would therefore indicates a low likelihood of overfitting. To further assess the robustness of our findings, SHAP values were computed on the external test dataset to examine whether the feature importance patterns observed in Study 1 remained consistent in an unseen sample (Study 2). In this external testing, success was defined as evaluation metrics exceeding the average values expected under random binary classification (i.e., AUC and balanced accuracy >0.5, F1 score >0.31 given unbalanced base rates). All analyses were conducted in Python using the scikit-learn (v1.5.2)^93^, SciPy (v1.13.1)^94^, and SHAP (v0.46.0)^50^ packages.

## Supplementary information

Supplementary information

Reporting_Summary_MJ

Checklist_Editorial_Policy_MJ

## Data Availability

The datasets generated and/or analyzed during the current study have been de-identified and anonymized and are publicly available on GitHub ([https://github.com/mgd-fchs/SHINE-DHI-effectiveness-prediction]). The social network datasets generated and/or analyzed during the current study are not publicly available due to their sensitive nature and concerns about the possibility of re-identifying individuals from social network data, but are available from the corresponding author on reasonable request.

## Code availability

All code used for pre-processing and analysis is provided on GitHub (https://github.com/mgd-fchs/SHINE-DHI-effectiveness-prediction) under the MIT License and can be freely accessed and reused in accordance with the license terms.
