# Supplementary material for: Predicting individual differences in digital alcohol intervention effectiveness through multimodal data
Source: NPJ Digit Med. 2026 Jan 27;9:170. doi: 10.1038/s41746-026-02356-4 (PMC12913919; doi:10.1038/s41746-026-02356-4)
Supplement: Supplementary file 1 — Supplementary information [file 41746_2026_2356_MOESM1_ESM.pdf]

The following document contains supplementary information for Fuchs et al. “Predicting Individual Differences in Digital Alcohol Intervention Effectiveness Through Multimodal Data”.

1- Theoretical Background

The following paragraphs motivate the choice of candidate features evaluated for predictability of individual differences, i.e., changes in drinking frequency, in response to the psychological distancing DHIs:

Drinking motives and expected effects of alcohol can further moderate intervention effectiveness[1]. For example, young adults who tend to drink to enhance mood or conform socially typically hold strong positive expectations about alcohol's effects (e.g., increased sociability, enjoyment, or social belonging)[2]. These positive expectations may in turn reinforce more favorable attitudes toward alcohol[3], amplify sensitivity to social pro-drinking norms[4], and reduce perceived self-control over one's alcohol consumption[5]. Conversely, individuals who expect negative health-related or impairing consequences from drinking typically exhibit less favorable attitudes toward drinking; are less sensitive to pro-drinking norms; and hold stronger intentions to reduce their drinking—possibly enhancing the effectiveness of distancing interventions in promoting alcohol control.

In parallel, a large body of neuroimaging research offers complementary insights into the neural mechanisms underlying health behaviors such as drinking[6] and individual differences in intervention-driven health behavior change[7,8]. Individual differences in brain activity related to reward processing and regulation, in response to alcohol cues, have been linked to differences in reactivity to alcohol cues and downstream drinking behavior. For instance, stronger activation in reward-related regions—such as the ventral striatum—in response to alcohol cues is associated with stronger cravings and a higher likelihood of alcohol consumption[9]. Increased neural reward sensitivity to alcohol cues may reduce the effectiveness of interventions that aim to decouple the link between cue reactivity (e.g., seeing a drink, experiencing a craving) and the subsequent behavior (drinking)[10]. Conversely, stronger activation in brain regions involved in regulation—such as the dorsolateral prefrontal cortex and anterior cingulate cortex—is linked to greater control over cravings triggered by alcohol cues[11,12]. These neural responses may facilitate the effective use of psychological distancing strategies by helping individuals create mental space from alcohol cues, which can ultimately lead to reduced drinking.

Further, neural mechanisms related to social influence on drinking may also serve as important moderators of intervention effectiveness, as research suggests they help explain how different individuals interpret and respond to peer-related cues and social norms around alcohol use. For example, stronger activation in brain regions involved in social cognition or mentalizing—such as the temporoparietal junction (TPJ), medial prefrontal cortex (mPFC), and posterior superior temporal sulcus (pSTS)[13]—in response to different peer contexts (e.g., drinking vs. non-drinking peers) may reflect heightened sensitivity to perceived social norms. This sensitivity can increase or decrease susceptibility to peer pressure and alcohol-related cues, depending on whether the peer influence promotes or discourages drinking[14]. Additionally, a measure of brain functional organization, resting-state network segregation, has been linked to self-regulatory capacity in behavior change interventions and shows promise as an indicator of individual differences in intervention responsiveness[15,16]. Building on this work, individuals with higher segregation at rest may also exhibit more effective neural decoupling from alcohol cues, supporting greater

potential for behavior change. Together, different neural measures have been proposed to explain how individuals approach alcohol cues and social influences on alcohol, which may explain individual differences in intervention-driven behavior change.

Individual differences in emotional and cognitive functioning can also relate to how young adults respond to psychological distancing interventions and drinking. For example, higher levels of trait anxiety or depression[17] may heighten emotional reactivity to alcohol cues, making it harder to mentally step back and engage in distancing strategies during moments of stress or craving. Similarly, poor emotion regulation[18] and attentional control may impair the ability to interrupt automatic responses to triggers and refocus on long-term goals, limiting effective distancing from alcohol cues[19]. Relatedly, impulsivity may increase susceptibility to immediate rewards, undermining the ability to pause and reflect in the presence of alcohol cues. Conversely, individuals with higher levels of mindfulness trait disposition—that is, a person's typical level of attention and awareness of present-moment experiences in daily life[20,21] or purpose in life[22] may be able to more effectively create distance from alcohol triggers. Similarly, individuals with stronger resistance to peer influence[23] and autonomy[24] may be less likely to conform to social influences on drinking, allowing for more effective adoption of distancing strategies. Together, these different psychological constructs tap into self-regulatory, emotional, and cognitive capacities that may help explain why some individuals may be better able than others to engage with and benefit from distancing interventions.

Beyond individual factors, a person's position within their social network also relates to alcohol use patterns[25]. Young adults who hold more central (vs. peripheral) positions in peer drinking networks—i.e., who are identified by others as drinking companions or heavy drinkers—are typically exposed to more drinking opportunities[26], are more closely connected to other heavy drinkers[27], and are surrounded with more social pressures to conform to drinking[24,28]. Together, these factors can reinforce drinking habits and possibly undermine intervention effectiveness. In addition to social network position, subjective perceptions of peer drinking approval and peer drinking behaviors can also play a role in shaping drinking behaviors[29]. Individuals who perceive their peers to drink heavily may be more likely to view drinking as normative and to align with those behaviors[30]. Specifically, when young adults perceive high peer approval of drinking, pro-drinking norms are more likely to be internalized, making behavior change more difficult[29,31]. Together, both individual differences in social network position and subjective beliefs about peer drinking may make it more difficult for some (or easier for others) to disengage from shared drinking behaviors, and potentially weaken or strengthen the impact of distancing interventions.

Multiple fields—including health psychology, behavioral science, neuroscience, and social network science—offer valuable perspectives on alcohol use and intervention responsiveness. However, few studies integrate these approaches to identify the most reliable predictors of behavior change. Comparing these predictors across disciplines is critical for developing targeted interventions, optimizing outcomes, and improving resource allocation, such as early identification of likely responders for tailored enrollment and pre-intervention planning.

2 - Sample Description

Inclusion/exclusion of participants

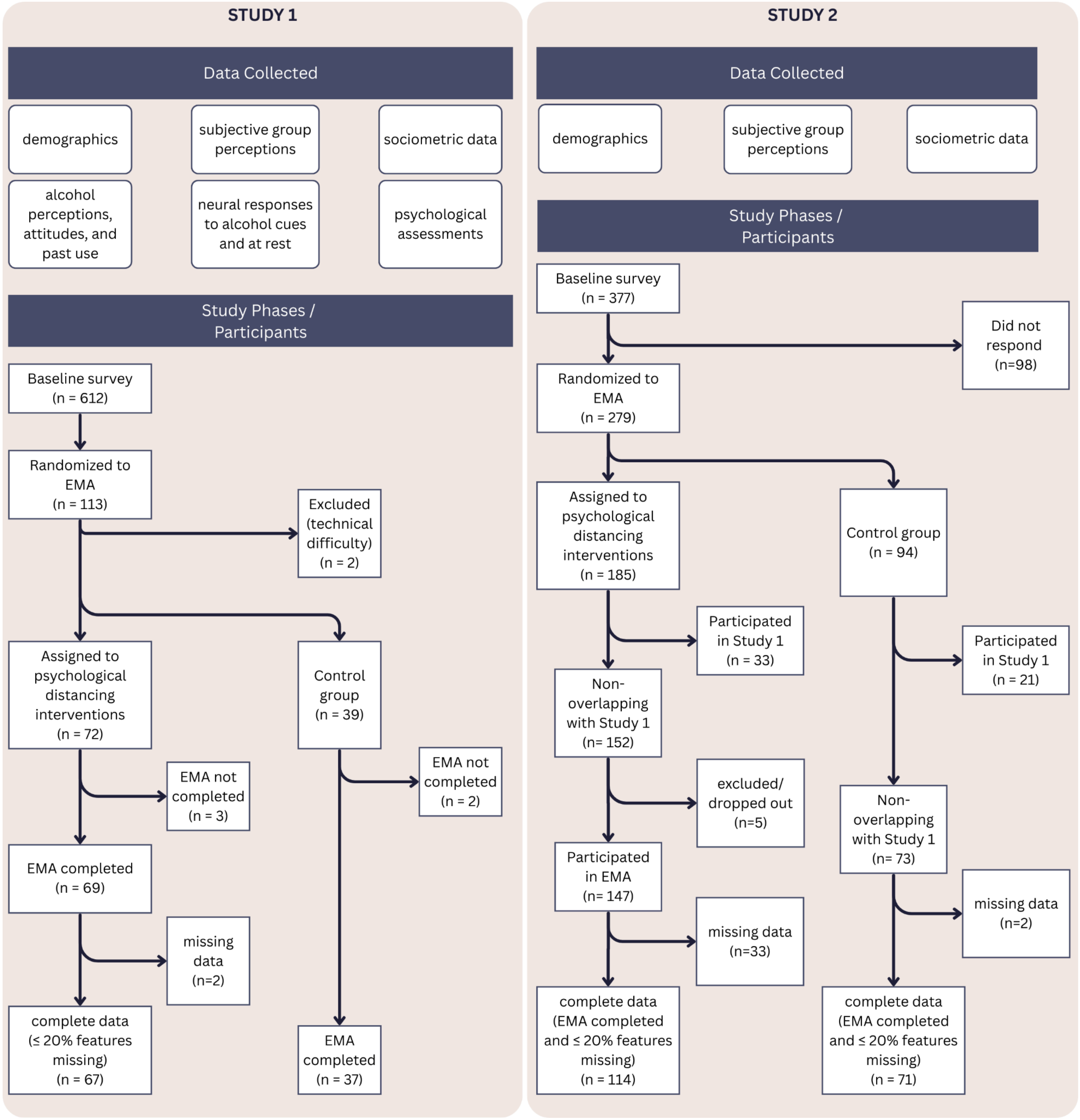

Figure S1. Participant flow chart across Study 1 and Study 2. The diagram illustrates the number of participants included or excluded at each stage of data collection. Data categories collected at each stage are also shown, including demographic, behavioral, psychological, social, and neuroimaging features.

Sample Descriptives: Study 1 & 2

Table S1. Sample descriptives for Study 1 and Study 2.

Characteristic	Study 1 (N = 67)	Study 2 (N = 114)
**Age, M, SD, (Min–Max)**	20.48, 1.76, (18–28)	20.15, 1.26, (18–25)
**Adherence (% of completed prompts), M, SD, (Min-Max)**	90.25, 12.62, (42.86-100.0)	82.07, 25.64, (1.79-100.0)
**Gender**		
Male	26 (38.8%)	23 (20.2%)
Female	41 (61.2%)	91 (79.8%)
Non-binary	0 (0.0%)	0 (0.0%)
**Race**		
white	37 (55.2%)	44 (38.6%)
Asian	21 (31.3%)	40 (35.1%)
Black or African American	2 (3.0%)	12 (10.5%)
Latino/a	2 (3.0%)	6 (5.3%)
Multiracial / Other	5 (7.5%)	8 (7.0%)
Missing	0 (0.0%)	2 (1.8%)
**College year**		
Year 1	18 (26.9%)	19 (16.7%)
Year 2	12 (17.9%)	31 (27.2%)
Year 3	19 (28.4%)	29 (25.4%)
Year 4	16 (23.9%)	31 (27.2%)
Year 5	2 (3.0%)	2 (1.8%)
Missing	0 (0.0%)	2 (1.8%)
**Household income**		
Median (Min-Max)	149.99k, (4.99k-200k)	149.99k, (4.99k-200k)
**Intervention condition**		
Mindfulness	37 (55.2%)	58 (50.9%)
Perspective taking	30 (45.8%)	56 (49.1%)
**Participants with >1 drinking occasion reduction**	15 (22.4%)	14 (12.3%)

Sample descriptives: mindful vs perspective-taking strategy

Table S2. Sample descriptives for Study 1 and Study 2 separated by intervention strategy (mindfulness and perspective). AUQ self-report baseline drinking data is not available for Study 2 (see Figure S1) and thus omitted.

	**Study 1**		**Study 2**	
**Intervention strategy**	**Mindfulness (N=37)**	**Perspective-taking (N=30)**	**Mindfulness (N=58)**	**Perspective-taking (N=56)**
**Adherence (% of completed prompts), M, (Min-Max)**	91%, (55%-100%)	90%, (43%-100%)	82%, (5%-100%)	78%, (2%-100%)
**Baseline drinking**				
baseline drinking amount AUQ self-report (drinks/occasion)	2.77, 1.66, (0.0-7.0)	3.10, 2.67, (0.0-12.0)	-	-
baseline drinking frequency AUQ self-report (drinking occasions/week)	0.60, 0.58, (0.0-2.5)	0.42, 0.51, (0.0-2.5)	-	-
**Gender**				
Women	16 (43.2%)	10 (33.3%)	10 (17.2%)	13 (23.2%)
Men	21 (56.8%)	20 (66.7%)	48 (82.8%)	43 (76.8%)
**Race/ethnicity**				
white	20 (54.1%)	17 (56.7%)	20 (34.5%)	24 (42.9%)
Asian	12 (32.4%)	9 (30.0%)	24 (41.4%)	16 (28.6%)
Black or African American	1 (2.7%)	1 (3.3%)	6 (10.3%)	6 (10.7%)
Latino/a	1 (2.7%)	1 (3.3%)	3 (5.2%)	3 (5.4%)
Multiracial / Other	3 (8.1%)	2 (6.7%)	4 (6.9%)	6 (10.7%)
Missing	0 (0.0%)	0 (0.0%)	1 (1.7%)	1 (1.8%)
**Age, M, SD, (Min–Max)**	20.7, 1.87, (18-28)	20.2, 2.61, (18-24)	20.2, 1.11, (18-22)	20.1, 1.41, (18-25)
**Participants with >1 drinking occasion Reduction**	10 (27.0%)	5 (16.7%)	8 (13.8%)	6 (10.7%)

Sample descriptives among responsive, non-responsive, and controls

Systematic differences between responsive and non-responsive individuals and between the control and active groups in Study 1 were examined. Of the 41 participants assigned to the control condition, 37 completed the EMA and had complete data. Chi-squared tests were used for categorical variables (gender, race), and independent-samples t-tests were used for continuous variables (self-reported drinking frequency, drinking amount, and age). All p-values were non-significant, indicating no statistically meaningful differences between the control and active groups on these baseline characteristics.

In Study 1, responsive individuals identified as white (73.3%), Asian (6.7%), Black/African American (6.7%), Latino/a/x (0.0%), and multiracial (13.3%). In Study 2, responsive participants identified as white (42.9%), Asian (28.6%), Black/African American (7.1%), Latino/a/x (0.0%), and multiracial (7.1%). In both studies, responders and non-responders did not differ significantly from the sample mean in terms of age. Study 1 participants considered responsive to the intervention were slightly higher drinkers at baseline, self-reporting a baseline drinking frequency of 0.75 drinking occasions per week and 3.0 drinks per occasion on their baseline alcohol use questionnaire (AUQ), compared to 0.25 occasions per week and 2.0 drinks per occasion in non-responders (see Supplementary Table S3). The same data on baseline drinking is not available for participants of Study 2 due to the difference in collected data modalities (Supplementary Figure S1). During the intervention, responders drank more frequently than non-responders on control weeks (p=0.021 in Study 1, p=0.83 in Study 2) but consumed significantly fewer drinks per occasion during active intervention weeks (p=0.001 Study 1, p<0.001 Study 2). During active intervention weeks, responders' drinking frequency dropped to a similar level as non-responsive individuals (p=0.74 Study 1, p=0.163 Study 2; Supplementary Tables S11 and 12, Supplementary Figures S9 and S10).

In addition to the AUQ self-reports used for prediction, we present another self-report measure for drinking behavior collected at baseline: The NIAAA drinking measures, adapted from the US National Institute on Alcohol Abuse and Alcoholism (NIAAA) Single Alcohol Screening Question (SASQ)[32], to compare Studies 1 and 2 (as AUQ data is only available for Study 1). In terms of NIAAA drinking amount and frequency measures, Study 1 responders and non-responders did not differ significantly in baseline drinking behavior (Mann-Whitney frequency: U = 488.5, p = 0.128; amount: U = 346.5, p = 0.773). The same pattern held in Study 2, with no significant baseline differences between responders and non-responders (frequency: U = 454.0, p = 0.869; amount: U = 473.5, p = 0.951). However, in Study 1, the intervention group reported higher baseline drinking frequency than the control group (p = 0.014), indicating a slight imbalance between study arms despite randomization.

Table S3. Sample descriptives for Study 1 and Study 2 by responsiveness status. The control group is included alongside measures of statistical significance to indicate whether they differ significantly from the Study 1 data (independent of responsiveness status). Baseline drinking data measured by the AUQ questionnaire is not available for Study 2 (see Figure S1) and thus omitted. NIAAA drinking measures are adapted from the US National Institute on Alcohol Abuse and Alcoholism (NIAAA) Single Alcohol Screening Question (SASQ), which has been extensively used for assessing alcohol use and drinking frequency[32].

	Study 1			Study 2		
**Characteristic**	**Responders (N=15)**	**Non-Responders (N=52)**	**Controls (N=37)**	**Responders (N=14)**	**Non-Responders (N=100)**	**Controls (N=71)**
**Adherence (% of completed prompts), M, SD, (Min-Max)**	93%, (68%-100%)	95%, (43%-100%)	93%, (13-100%)	92%, (45%-100%)	93%, (2%-100%)	89.3%, (7.1%-100.0%)
**Baseline drinking**						
baseline drinking amount AUQ self-report (drinks/occasion)	3.0, 1.86, (0.0-8.0)	2.0, 2.25, (0.0-12.0)	3.1, 2.0, (0.0-9.0), *p=0.677*	-	-	-
baseline drinking frequency AUQ self-report (drinking occasions/week)	0.75, 0.33, (0.0-1.0)	0.25, 0.60, (0.0 - 2.5)	1.9, 1.4, (0.0-6.0), *p=0.089*	-	-	-
Baseline drinking amount in the past 6 months (NIAAA)	3.6, 1.4, (2.0-7.5)	3.8, 1.9, (1.0-10.0)	4.36, 1.75, (2.0-7.5) *p = 0.111*	4.5, 2.4, (1.0-7.5)	3.7, 1.8, (1.0-7.0)	3.6, 1.9, (1.0-7.5) *p = 0.982*
Baseline drinking frequency in the past 6 months (NIAAA)	84.1, 55.1, (12.0-182.0)	59.9, 41.0, (1.5-182.0)	94.5, 65.3, (7.0-286.0) *p=0.014*	78.3, 100.1, (12.0-365.0)	69.8, 57.0, (1.5-182.0)	97.53, 65.3, (1.5-286.0)
**Gender**			$\chi^2(1) = 0.01$, *p = 0.943*			$\chi^2(1) = 0.003$, *p = 0.957*
Men	8 (53.3%)	18 (34.6%)	15 (40.5%)	4 (28.6%)	19 (19.0%)	15 (21.1%)
Women	7 (46.7%)	34 (65.4%)	22 (59.5%)	10 (71.4%)	81 (81.0%)	39 (54.6%)
Missing	0 (0.0%)	0 (0.0%)	0 (0.0%)	0 (0.0%)	0 (0.0%)	17 (23.9%)
**Race/ethnicity**			$\chi^2(4) = 4.19$, *p = 0.381*			$\chi^2(4) = 3.96$, *p = 0.994*
White	11 (73.3%)	26 (50.0%)	19 (51.4%)	6 (42.9%)	38 (38.0%)	25 (35.2%)
Asian	1 (6.7%)	20 (38.5%)	11 (29.7%)	4 (28.6%)	36 (36.0%)	17 (23.9%)
Black or African American	1 (6.7%)	1 (1.9%)	0 (0%)	1 (7.1%)	11 (11.0%)	3 (4.2%)
Latino/a	0 (0%)	2 (3.8%)	3 (8.1%)	0 (0%)	6 (6.0%)	2 (2.8%)
Multiracial / Other	2 (13.3%)	3 (5.8%)	4 (10.8%)	1 (7.1%)	7 (7.0%)	7 (9.9%)
Missing	0 (0%)	0 (0%)	0 (0%)	0 (0%)	2 (2.0%)	17 (23.9%)
**Age, M, SD, (Min–Max)**	20.5, 1.55, (18-23)	20.5, 1.83, (18-28)	20.32, 1.58, (18-26), *p=0.575*	20.5, 1.16, (18-22)	20.1, 1.28, (18-25)	20.5, 3.46, (18.0-42.0)

Adherence by responsiveness

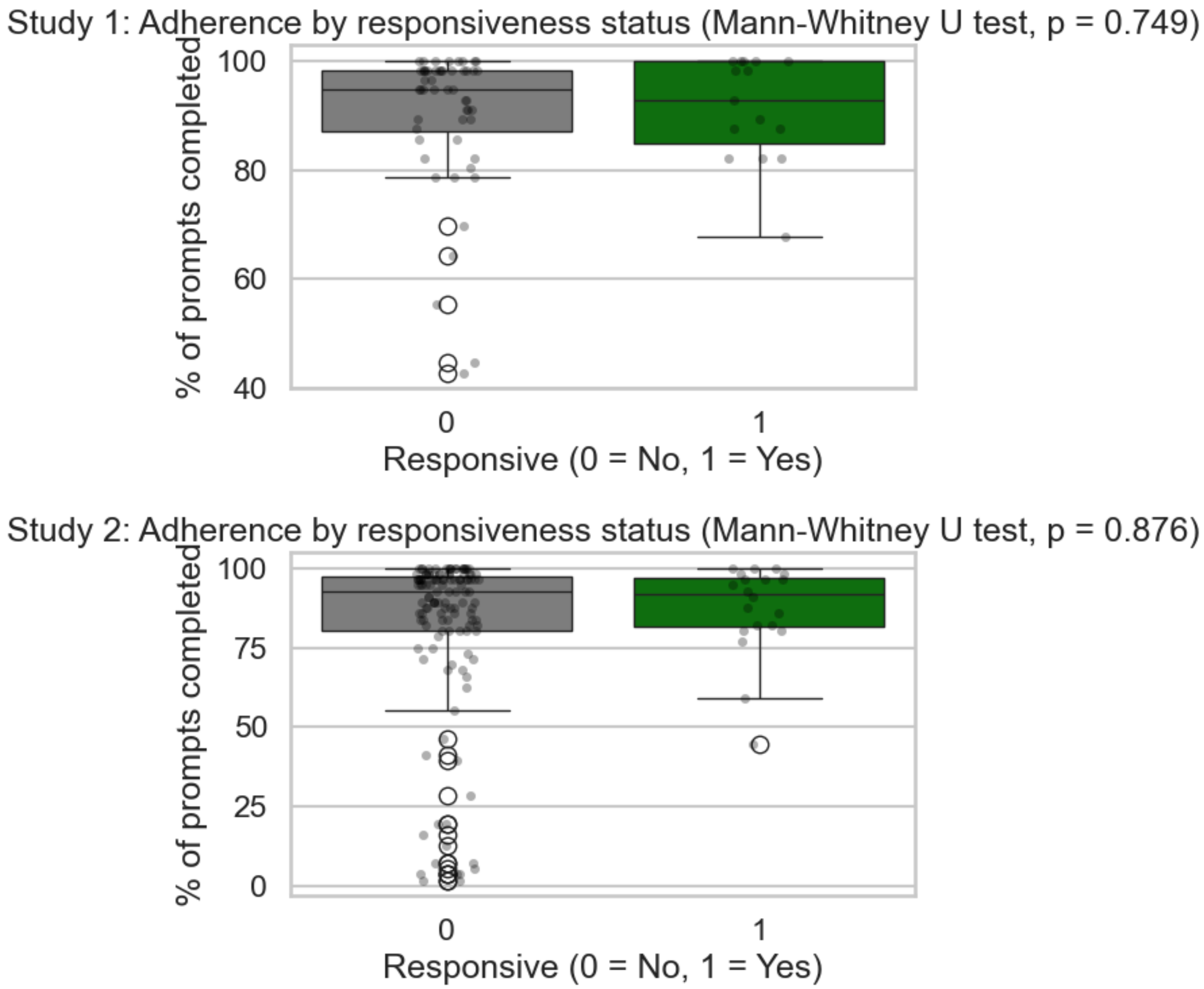

Figure S2. Adherence (in % of alcohol prompts completed) by responsiveness status in both studies. The distribution of adherence levels is not statistically significantly different between responders and non-responders ($p>0.7$ in both studies). There is, however, a larger variance in adherence for the non-responders, including some outliers that have very low (<10% adherence).

3 - Measures and Scales

To predict individual differences in intervention effectiveness (RQ1), we considered baseline data across six distinct domains—(1) baseline alcohol use and cognitions; (2) psychological assessments; (3) sociometric nominations; (4) neural responses to alcohol, social cues and at rest; (5) subjective drinking perceptions of peers, i.e., actual group members; and (6) demographics. In the following section, we describe how each feature category was collected and operationalized. Next, we also present the full list of variables included in modeling.

Baseline alcohol use and cognitions

Participants' alcohol-related behaviors, motivations, and expectations were assessed using a combination of validated self-report instruments, yielding 16 total variables. These included past 6-month drinking frequency, typical quantity consumed per occasion, and future drinking intentions, measured using the Alcohol Use Questionnaire (AUQ)[33]. Binge drinking attitudes, the intention to engage in binge drinking, and self-efficacy to avoid it were adapted from Ref.[34]. Motivations for alcohol use were measured via the Drinking Motives Questionnaire (DMQ), which included subscales for drinking to cope, for social reasons, for enhancement, and conformity[35]. Alcohol-related expectancies were captured using the Drinking Expectancy Questionnaire—Revised Adolescent Version (DEQ-RA)[36], assessing beliefs about the likelihood of outcomes such as tension reduction, increased confidence, sexual enhancement, cognitive impairment, and negative consequences.

Psychological assessments

We assessed a broad set of validated self-report instruments that captured emotional, cognitive, and other psychological traits relevant to alcohol use and intervention response. These included the State-Trait Anxiety Inventory (STAI) to assess trait anxiety[37] and the Center for Epidemiologic Studies Depression Scale (CES-D)[38] for depressive symptom severity. Cognitive control and attentional flexibility were assessed using the Attentional Control Scale (ACS)[39], while impulsivity was measured via the Barratt Impulsiveness Scale (BIS)[40], including subscales for motor, attentional, and non-planning impulsivity. Emotion regulation was evaluated through the Difficulties in Emotion Regulation Scale (DERS)[41], which includes subcomponents such as emotional clarity, impulse control, access to regulation strategies, and goal-directed behavior under distress. Additional constructs included the Mindful Attention Awareness Scale (MAAS) to measure present-moment awareness[42]; assessing individual differences in susceptibility to peer pressure[43], the Purpose in Life Scale (PILS)[44], measuring sense of life purpose; the Interpersonal Autonomy Scale (IAS)[45], assessing social inhibition and autonomy; and a short form of the UCLA Loneliness Scale[46], capturing feelings of loneliness. Together, these scales contributed 20 features across various affective and cognitive domains.

Peer drinking perceptions

We assessed participants' perceptions of their peers' alcohol-related behaviors and attitudes using five measures designed to capture how individuals believed members of their social group thought about and engaged in alcohol use. These measures, adapted from Ref.[47], included perceived peer approval of alcohol use, which reflected the extent to which participants believed their peers approved of general alcohol consumption, and perceived peer approval of binge drinking, which captured beliefs about how much peers approved of heavy drinking. We also measured perceived peer drinking norms, defined as participants' sense of shared expectations around alcohol use within their group. In addition to these normative beliefs, we derived two behavioral features based on participants' perceptions of high-profile drinkers within their group. Participants were asked to nominate the individuals they believed drank the most. For each participant, we then calculated the average perceived drinking frequency and the average

typical drinking amount per occasion for those nominated peers. These two features were intended to capture perceived high-risk drinking within their social group.

Sociometric peer nominations

To assess participants' positions within their social group, we used a sociometric nomination approach[48] that captured how individuals were perceived by their peers across multiple social dimensions. At baseline, participants completed a social network survey in which they nominated fellow group members in response to eight survey prompts[49]. These included: "Who do you like the most?", "Who are you closest to?", "Who did you interact with most recently?", "Who drinks the most?", "Who drinks the least?", "Who is the most influential?", "Who are the leaders in the group?", and "Who would you go to when something bad (or good) happens?" For each item, participants could nominate as many peers from their group as they wanted, with no limit.

We then computed in-degree scores, defined as the number of incoming nominations each participant received on each item[50]. For example, a participant with a high in-degree on the "drinks the most" item was frequently nominated by peers as a heavy drinker, while a high in-degree on the "leader" item indicated they were widely recognized as a group leader. These eight sociometric features served as objective network-based indicators of participants' social network position within their peer group.

Formally, the in-degree for participant v_i was calculated as:

$$In\text{-}degree(v_i) = \sum_{j=1}^{N} A_{ji}$$

where $A_{ji} = 1$ if participant j nominated participant i, 0 otherwise, and N denotes the total number of participants. In-degree thus captures how frequently an individual was nominated by their peers for a certain item (e.g., perceived as frequent drinkers).

Neural responses to alcohol cues, peer faces, and at rest

Neural features were derived from functional MRI (fMRI) data collected during three scans: an alcohol cue reactivity task, a face-viewing task, and a resting-state scan. Detailed fMRI task procedures and pre-processing are provided in the SHINE protocol[49]. During the alcohol cue reactivity task, participants viewed alcohol-related and neutral images (e.g., water). For trials used in this analysis, participants were instructed to simply observe the images. We extracted activity in reward-related regions, the ventral striatum, a key reward-processing region[9], and regulation regions associated with 'top-down' regulation of alcohol cue reactivity and cravings[11,12]. Regions of interest (ROIs) were defined using meta-analytic maps from a meta-analytic metabase, Neurosynth[51], for "ventral striatum," "reward," and "emotion regulation," and activation was measured by contrasting responses to alcohol cues versus rest.

In the face-viewing fMRI task[49], participants passively viewed images of peers from their social group. Drawing on related work on social influence mechanisms[14], we extracted neural

responses to faces of peers with whom one drank more vs. less frequently, in regions related to mentalizing—associated with the ability to understand others' thoughts and intentions[13]. This contrast captured social cognitive processes linked to peer influence, which prior research has associated with individual differences in sensitivity to social influence on drinking behavior[14]. Mentalizing ROIs were defined using Neurosynth meta-analytic maps[51]. Beyond task-based activation, we also included a resting-state measure of network segregation[15,16], which reflects the extent to which functional brain networks are organized into distinct and specialized systems at rest. In total, five neural features were extracted across the three scan types, capturing both task-evoked brain responses and intrinsic connectivity patterns relevant to alcohol use, social cognition, and self-regulation[51]. For full details on imaging acquisition and preprocessing, see the study protocol[49].

Demographic variables

Demographics measures included age, gender, race, college year, and annual family income.

All Features

Table S4. Full list of variables included in modeling across the six data domains. Features that were dropped due to high correlation with other features in the same categories (correlation coefficient > 0.8 are indicated in red).

Predictor Category	Demographics	Baseline alcohol use and cognitions	Psychological assessments	Peer drinking perceptions	Sociometric peer centrality nominations	Neural responses to alcohol, social cues and at rest
# features	5	16	20	5	8	8
	age (n=1)	alcohol attitudes (n=2)	State-Trait Anxiety (STAI) (n=1)	Perceived peer drinking frequency (n=1)	In-degree centrality: “like” network (n=1)	ROI activity in brain regions associated with reward during exposure to alcohol cues vs. rest (n = 1)
	gender (n=1)	drinking expectancy (DEQ) (n=5)	Attentional Control(ACS) (n=3) ACS_focus (dropped)	Perceived peer drinking amount (n=1)	In-degree centrality: “drinks least” network (n=1)	Whole-brain functional connectivity metric of segregation during rest (n = 1)
	Race (n=1)	drinking Motives (DMQ) (n=4)	Depression (CES-D) (n=1)	Perceived peer drinking frequency (n=1)	In-degree centrality: “drinks most” network (n=1)	ROI activity in predefined reward-related brain regions (n = 1).
	college year (n=1)	Alcohol use (AUQ) (n=3)	Mindful attention (MAAS), (n=1)	Perceived peer drinking approval (n=1)	In-degree centrality: “closest to” network , (n=1)	ROI activity in brain regions associated with mentalizing during exposure to drinking vs. non-drinking peer faces (n = 1)
	Income (n=1)	Binge Drinking (intent and efficacy), n=2	Resistance to peer influence (RPI) (n=1)	Perceived group of alcohol attitudes, n=1	In-degree centrality: “influential” network (n=1)	ROI activity in the ventral striatum during exposure to alcohol cues vs. rest (n = 1)
			Interpersonal Autonomy (IAS), (n=1)	Perceived group binge drinking attitudes, n=1	In-degree centrality: “go to for support when feeling good” network (n=1)	ROI activity in brain regions associated with cognitive control during exposure to alcohol cues vs. rest (n=1) (dropped)
			Purpose in Life, (n=1)		In-degree centrality:“go to when bad” network (n=1) (dropped)	ROI activity in brain regions associated with craving during exposure to alcohol cues vs. rest (n=1) (dropped)
			Loneliness (ULS-4), (n=1)		In-degree: “leaders” k (n=1) (dropped)	ROI activity in brain regions associated with emotion regulation during exposure to alcohol cues vs. rest (n=1) (dropped)
			Difficulties in Emotion Regulation (DERS), (n=6) DERS_strategies (dropped)			

Predictor Category	Demographics	Baseline alcohol use and cognitions	Psychological assessments	Peer drinking perceptions	Sociometric peer centrality nominations	Neural responses to alcohol, social cues and at rest
# features	5	16	20	5	8	8
			Impulsiveness (BIS), (n=4)<br>BIS_attention_total (dropped)			

4 - Model Configuration

Table S5. Hyperparameter values used in nested CV grid search for all four model types

Model Type	Parameter	Values
Random Forest	Number of trees (*n_estimators*)	50
	Maximum tree depth (*max_depth*)	3, 5
	Minimum samples to split a node (*min_samples_split*)	2, 4, 8
	Minimum samples per leaf node (*min_samples_leaf*)	2, 3, 5
Logistic Regression	Penalty (*penalty*)	l2, None
	Solver (*solver*)	lbfgs, saga
	Maximum iterations (*max_iter*)	500, 1000
	Regularization (*C*)	0.01, 0.1, 1
SVM (radial)	Kernel type (*kernel*)	rbf
	Kernel coefficient (*gamma*)	"scale", 1e-3, 1e-2, 1e-1
	Regularization (*C*)	1e-3, 1e-2, 1e-1, 1, 10
SVM (linear)	Kernel type (*kernel*)	linear
	Regularization (*C*)	1e-2, 1e-1, 1, 10

5 - Drinking Fluctuations in the Control Group

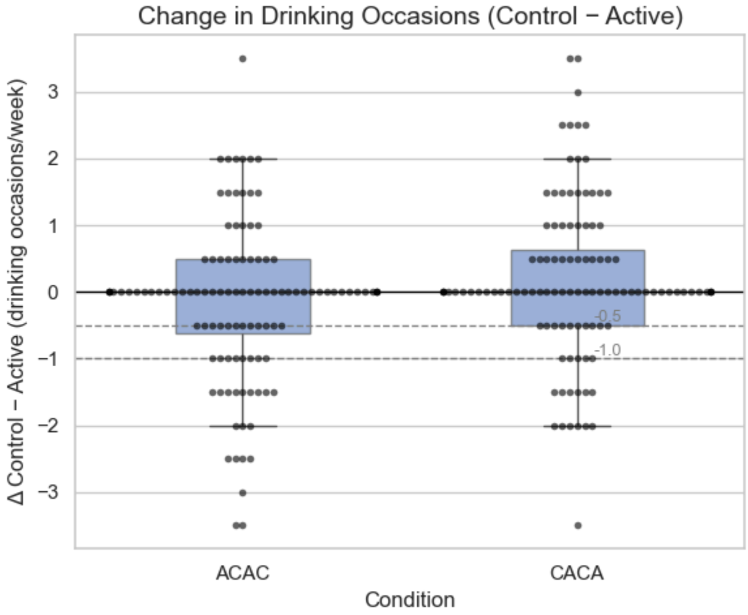

Figure S3. Average change in weekly drinking occasions for control group participants. Control group participants were randomly allotted to "control" (C) and "active" (A) weeks, and the difference between these weeks was computed in the same way as for the actual intervention participants. Thresholds -0.5, -1.0, and -2.0 are indicated in the figure. Negative values reflect that a participant reduced their drinking in active weeks compared to control weeks.

Table S6. Average change in weekly drinking occasions for control group participants. Control group participants were randomly allotted to "inactive" and "active" weeks, and the difference between these weeks was computed in the same way as for the actual intervention participants. The resulting difference gives an indication of the natural fluctuation in drinking occasions between weeks that we would expect in the absence of the psychological distancing interventions.

**Condition**	**Mean Δ(control−active)**	**SD**
ACAC (on-off-on-off)	*-0.18*	*1.21*
CACA (off-on-off-on)	*0.18*	*1.21*

6 - Supplementary Results

Partial dependence plots

Partial dependence plots (PDPs) are used to explore the relationships between key features and model predictions in the model development set from Study 1. These plots enable the identification of non-linear effects, threshold patterns, and interactions between features by showing the marginal effects of up to two features on the output of the ML model[52]. For the 'PEER' features, PDPs shown in Figure S1 illustrate how perceived peer behavior influences the prediction of intervention responsiveness. For *perceived peer drinking amount*, a threshold effect can be observed: once perceived consumption exceeds approximately three drinks per occasion, the likelihood of predicting a positive intervention response (shown on the y-axis) declines sharply. Similarly, *perceived peer drinking frequency* exhibits a non-linear relationship with intervention effectiveness prediction: Participants who perceived their peers to drink on 60–80 occasions per year are most likely to receive a positive effectiveness prediction, whereas lower perceived peer drinking frequencies were associated with reduced likelihood of positive prediction, and the likelihood of positive response plateaus for more frequent perceived drinking.

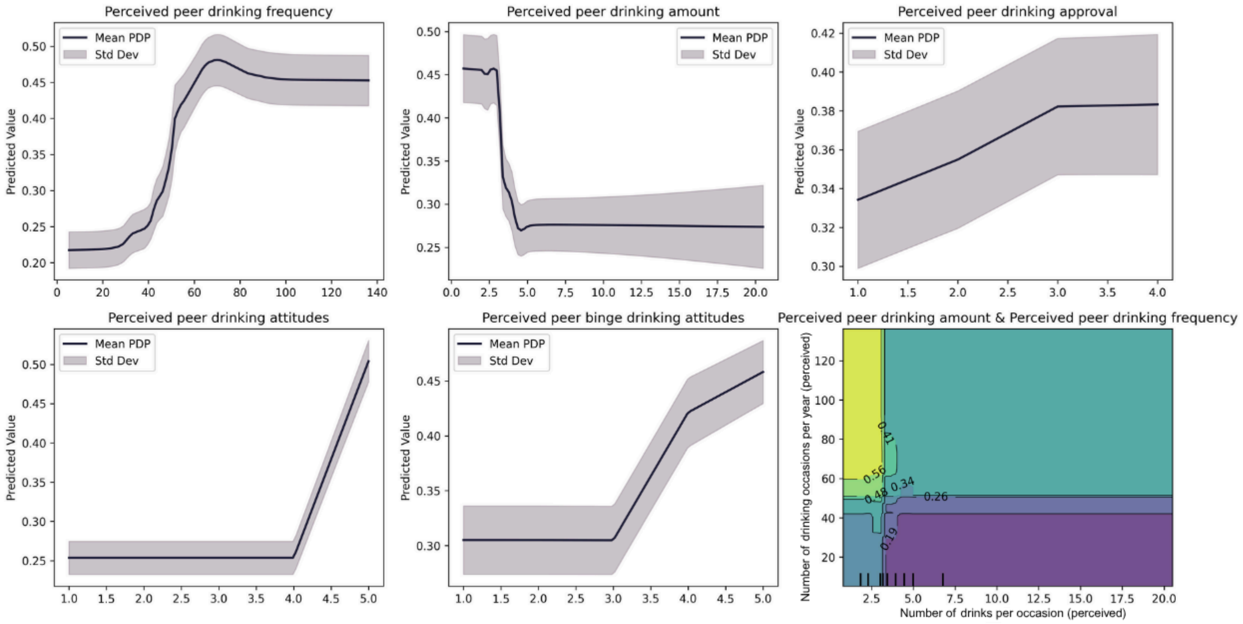

Figure S4. Partial dependence plots and a 2D partial dependence interaction plot for the most predictive peer drinking perception features in Study 1. The top and middle left panels show univariate PDPs for perceived peer drinking frequency, peer drinking amount, peer pressure, group attitudes, and group attitudes toward binge drinking. These plots display the average predicted probability of being classified as "responsive", i.e., effective intervention (class 1), across the range of each feature, with shaded areas representing standard deviations across 100 model repetitions. The bottom right panel displays a 2D PDP interaction plot for peer drinking frequency and amount, illustrating how the joint effect of these two features influences the predicted likelihood of intervention effectiveness. Color intensity reflects the model's predicted probability of being a responder, with lighter regions corresponding to higher predicted probabilities.

Validation results for all model types

Table S7. Full performance metrics across four different prediction models: Random forest, logistic regression (ElasticNet penalty), linear SVM, and SVM with radial basis function. Results are shown for all feature categories evaluated in the nested CV framework. Each metric is reported with its 95% confidence interval, computed across 100 outer loop repetitions. The PEER feature category is highlighted in blue, and results for the main RF model presented in the main manuscript are bolded. The expected performance of a random classifier varies by metric: it is 0.50 for AUC, balanced accuracy, and PR-AUC, but shifts due to class imbalance (23% positive class) to 0.31 for F1 score, 0.23 for sensitivity, and 0.77 for specificity. These baseline values provide reference points to assess whether model performance reliably exceeds chance levels.

Feature Category	Model	AUC	F1	Balanced Accuracy	Sensitivity	Specificity	PR-AUC	PPV	NPV
D	RF	0.485 (0.461–0.509)	0.233 (0.205–0.261)	0.505 (0.487–0.522)	0.234 (0.204–0.264)	0.775 (0.752–0.798)	0.291 (0.267–0.315)	0.245 (0.204–0.291)	0.762 (0.753–0.772)
	LR	0.456 (0.433–0.479)	0.266 (0.240–0.292)	0.477 (0.457–0.496)	0.404 (0.354–0.454)	0.549 (0.509–0.590)	0.304 (0.274–0.333)	0.219 (0.184–0.257)	0.75 (0.72–0.78)
	SVM (linear)	0.461 (0.434–0.488)	0.168 (0.136–0.200)	0.483 (0.465–0.501)	0.226 (0.180–0.272)	0.740 (0.696–0.784)	0.275 (0.253–0.297)	0.214 (0.156–0.282)	0.750 (0.737–0.763)
	SVM (radial)	0.489 (0.460–0.518)	0.030 (0.012–0.048)	0.488 (0.477–0.499)	0.030 (0.013–0.047)	0.946 (0.920–0.972)	0.298 (0.272–0.325)	0.147 (0.048–0.341)	0.755 (0.749–0.761)
ALC	RF	0.678 (0.657–0.699)	0.216 (0.175–0.256)	0.541 (0.522–0.560)	0.186 (0.148–0.224)	0.896 (0.878–0.914)	0.403 (0.372–0.433)	0.359 (0.275–0.450)	0.780 (0.772–0.789)
	LR	0.562 (0.518–0.606)	0.339 (0.287–0.390)	0.529 (0.492–0.566)	0.460 (0.387–0.533)	0.598 (0.547–0.649)	0.457 (0.409–0.506)	0.314 (0.254–0.378)	0.562 (0.518–0.606)
	SVM (linear)	0.360 (0.316–0.404)	0.349 (0.303–0.395)	0.517 (0.488–0.547)	0.565 (0.487–0.643)	0.470 (0.397–0.543)	0.278 (0.244–0.311)	0.299 (0.244–0.360)	0.718 (0.676–0.760)
	SVM (radial)	0.353 (0.312–0.394)	0.346 (0.293–0.399)	0.548 (0.518–0.579)	0.495 (0.416–0.574)	0.602 (0.530–0.674)	0.291 (0.257–0.324)	0.332 (0.261–0.413)	0.760 (0.733–0.787)
PSY	RF	0.536 (0.512–0.559)	0.121 (0.090–0.152)	0.490 (0.475–0.505)	0.102 (0.075–0.129)	0.877 (0.856–0.899)	0.302 (0.279–0.324)	0.206 (0.140–0.284)	0.757 (0.750–0.764)
	LR	0.629 (0.609–0.650)	0.374 (0.348–0.400)	0.574 (0.555–0.593)	0.450 (0.410–0.490)	0.698 (0.674–0.722)	0.373 (0.349–0.397)	0.332 (0.296–0.370)	0.797 (0.784–0.809)
	SVM (linear)	0.501 (0.466–0.536)	0.328 (0.294–0.362)	0.543 (0.519–0.567)	0.416 (0.366–0.466)	0.671 (0.642–0.699)	0.302 (0.274–0.330)	0.296 (0.254–0.341)	0.780 (0.764–0.796)
	SVM (radial)	0.405 (0.381–0.428)	0.164 (0.125–0.204)	0.514 (0.496–0.532)	0.206 (0.151–0.261)	0.822 (0.784–0.860)	0.222 (0.207–0.237)	0.278 (0.190–0.382)	0.764 (0.751–0.777)
PEER	**RF**	**0.867 (0.853–0.881)**	**0.548 (0.510–0.586)**	**0.710 (0.689–0.732)**	**0.504 (0.458–0.550)**	**0.917 (0.902–0.932)**	**0.714 (0.685–0.744)**	**0.655 (0.593–0.717)**	**0.860 (0.849–0.871)**
	LR	0.790 (0.769–0.811)	0.556 (0.529–0.584)	0.719 (0.698–0.739)	0.670 (0.633–0.707)	0.767 (0.745–0.790)	0.575 (0.539–0.610)	0.474 (0.437–0.512)	0.884 (0.872–0.896)
	SVM (linear)	0.775 (0.745–0.806)	0.566 (0.539–0.594)	0.729 (0.708–0.749)	0.694 (0.656–0.732)	0.763 (0.743–0.783)	0.568 (0.533–0.602)	0.478 (0.444–0.513)	0.892 (0.880–0.904)
	SVM (radial)	0.824 (0.807–0.841)	0.552 (0.525–0.578)	0.720 (0.699–0.740)	0.658 (0.615–0.701)	0.781 (0.761–0.801)	0.624 (0.592–0.657)	0.485 (0.446–0.524)	0.886 (0.873–0.899)
SOC	RF	0.590 (0.565–0.614)	0.274 (0.240–0.308)	0.530 (0.510–0.550)	0.292 (0.252–0.332)	0.769 (0.742–0.796)	0.344 (0.314–0.373)	0.283 (0.233–0.337)	0.778 (0.767–0.789)
	LR	0.633 (0.611–0.654)	0.390 (0.369–0.411)	0.578 (0.559–0.597)	0.594 (0.553–0.635)	0.562 (0.539–0.586)	0.356 (0.332–0.380)	0.298 (0.272–0.324)	0.822 (0.808–0.837)
	SVM (linear)	0.538	0.400	0.586	0.640	0.531	0.327	0.299	0.843

Feature Category	Model	AUC	F1	Balanced Accuracy	Sensitivity	Specificity	PR-AUC	PPV	NPV
		(0.502–0.574)	(0.377–0.423)	(0.564–0.607)	(0.590–0.690)	(0.497–0.566)	(0.294–0.360)	(0.268–0.332)	(0.823–0.863)
	SVM (radial)	0.388 (0.366–0.410)	0.103 (0.069–0.136)	0.510 (0.499–0.522)	0.162 (0.105–0.219)	0.859 (0.813–0.905)	0.206 (0.193–0.219)	0.264 (0.150–0.417)	0.775 (0.764–0.786)
NR	RF	0.579 (0.556–0.602)	0.258 (0.225–0.291)	0.529 (0.510–0.548)	0.256 (0.221–0.291)	0.802 (0.777–0.826)	0.319 (0.297–0.341)	0.288 (0.237–0.344)	0.775 (0.766–0.784)
	LR	0.461 (0.440–0.481)	0.232 (0.207–0.257)	0.450 (0.432–0.468)	0.362 (0.313–0.411)	0.539 (0.498–0.579)	0.254 (0.229–0.279)	0.197 (0.163–0.234)	0.730 (0.699–0.758)
	SVM (linear)	0.504 (0.479–0.530)	0.307 (0.283–0.331)	0.475 (0.457–0.493)	0.632 (0.564–0.700)	0.318 (0.265–0.371)	0.262 (0.242–0.282)	0.225 (0.193–0.258)	0.744 (0.719–0.769)
	SVM (radial)	0.506 (0.483–0.530)	0.356 (0.339–0.373)	0.508 (0.497–0.519)	0.794 (0.733–0.855)	0.221 (0.157–0.286)	0.271 (0.251–0.290)	0.242 (0.214–0.272)	0.767 (0.741–0.792)
D-ALC	RF	0.668 (0.644–0.693)	0.203 (0.165–0.241)	0.539 (0.522–0.556)	0.162 (0.129–0.195)	0.916 (0.902–0.929)	0.402 (0.373–0.431)	0.375 (0.291–0.463)	0.779 (0.771–0.786)
	LR	0.530 (0.487–0.573)	0.310 (0.263–0.357)	0.529 (0.502–0.556)	0.380 (0.319–0.441)	0.678 (0.624–0.732)	0.464 (0.418–0.510)	0.321 (0.254–0.397)	0.754 (0.716–0.784)
	SVM (linear)	0.386 (0.349–0.423)	0.351 (0.306–0.395)	0.526 (0.501–0.551)	0.590 (0.510–0.670)	0.462 (0.386–0.538)	0.299 (0.265–0.334)	0.305 (0.249–0.367)	0.753 (0.709–0.797)
	SVM (radial)	0.347 (0.309–0.385)	0.252 (0.201–0.303)	0.517 (0.493–0.541)	0.410 (0.325–0.495)	0.624 (0.545–0.703)	0.262 (0.232–0.292)	0.304 (0.222–0.400)	0.729 (0.707–0.751)
D-PSY	RF	0.525 (0.500–0.550)	0.147 (0.115–0.179)	0.513 (0.499–0.526)	0.124 (0.095–0.153)	0.901 (0.883–0.919)	0.302 (0.280–0.325)	0.282 (0.202–0.372)	0.768 (0.762–0.774)
	LR	0.589 (0.568–0.610)	0.326 (0.296–0.356)	0.546 (0.527–0.565)	0.410 (0.365–0.455)	0.681 (0.655–0.708)	0.349 (0.323–0.374)	0.300 (0.261–0.342)	0.781 (0.769–0.794)
	SVM (linear)	0.473 (0.443–0.502)	0.311 (0.278–0.345)	0.534 (0.511–0.557)	0.420 (0.363–0.477)	0.648 (0.615–0.681)	0.269 (0.247–0.291)	0.285 (0.239–0.332)	0.783 (0.765–0.801)
	SVM (radial)	0.428 (0.403–0.452)	0.066 (0.039–0.092)	0.494 (0.484–0.505)	0.086 (0.048–0.124)	0.903 (0.866–0.940)	0.245 (0.228–0.262)	0.228 (0.106–0.407)	0.747 (0.739–0.754)
D-PEER	RF	0.835 (0.817–0.852)	0.458 (0.421–0.496)	0.656 (0.638–0.675)	0.400 (0.362–0.438)	0.912 (0.898–0.927)	0.644 (0.611–0.677)	0.588 (0.526–0.652)	0.832 (0.823–0.840)
	LR	0.738 (0.715–0.761)	0.479 (0.449–0.508)	0.659 (0.638–0.681)	0.572 (0.533–0.611)	0.747 (0.724–0.770)	0.562 (0.529–0.594)	0.414 (0.376–0.453)	0.851 (0.838–0.863)
	SVM (linear)	0.717 (0.686–0.748)	0.482 (0.455–0.510)	0.663 (0.642–0.684)	0.602 (0.561–0.643)	0.724 (0.700–0.749)	0.538 (0.500–0.575)	0.406 (0.368–0.445)	0.858 (0.845–0.871)
	SVM (radial)	0.722 (0.688–0.756)	0.417 (0.382–0.452)	0.632 (0.610–0.653)	0.474 (0.426–0.522)	0.789 (0.769–0.810)	0.531 (0.495–0.567)	0.413 (0.366–0.462)	0.833 (0.821–0.846)
D-SOC	RF	0.593 (0.569–0.617)	0.228 (0.193–0.263)	0.534 (0.517–0.551)	0.204 (0.169–0.239)	0.864 (0.842–0.885)	0.360 (0.333–0.387)	0.319 (0.250–0.395)	0.777 (0.770–0.785)
	LR	0.554 (0.528–0.580)	0.335 (0.309–0.361)	0.541 (0.519–0.562)	0.476 (0.429–0.523)	0.605 (0.576–0.634)	0.319 (0.295–0.343)	0.274 (0.240–0.309)	0.791 (0.776–0.806)
	SVM (linear)	0.491 (0.455–0.527)	0.370 (0.343–0.398)	0.570 (0.550–0.590)	0.574 (0.519–0.629)	0.566 (0.528–0.603)	0.275 (0.249–0.301)	0.292 (0.256–0.331)	0.829 (0.811–0.847)
	SVM (radial)	0.451 (0.429–0.472)	0.045 (0.023–0.066)	0.497 (0.489–0.506)	0.050 (0.025–0.075)	0.945 (0.922–0.968)	0.247 (0.229–0.264)	0.221 (0.090–0.425)	0.761 (0.757–0.765)
D-NR	RF	0.525 (0.502–0.547)	0.203 (0.171–0.235)	0.521 (0.506–0.537)	0.176 (0.146–0.206)	0.867 (0.846–0.888)	0.327 (0.304–0.350)	0.292 (0.229–0.364)	0.771 (0.764–0.778)
	LR	0.425 (0.406–0.444)	0.262 (0.239–0.286)	0.467 (0.450–0.485)	0.430 (0.374–0.486)	0.505 (0.456–0.554)	0.296 (0.264–0.329)	0.214 (0.177–0.254)	0.739 (0.706–0.770)

Feature Category	Model	AUC	F1	Balanced Accuracy	Sensitivity	Specificity	PR-AUC	PPV	NPV
	SVM (linear)	0.493 (0.464–0.522)	0.302 (0.280–0.325)	0.468 (0.452–0.483)	0.620 (0.554–0.686)	0.315 (0.259–0.371)	0.300 (0.276–0.323)	0.220 (0.189–0.254)	0.697 (0.657–0.737)
	SVM (radial)	0.525 (0.504–0.546)	0.346 (0.325–0.367)	0.494 (0.488–0.501)	0.868 (0.807–0.929)	0.121 (0.064–0.177)	0.346 (0.324–0.369)	0.236 (0.212–0.261)	0.733 (0.705–0.760)
ALC-PSY	RF	0.631 (0.605–0.658)	0.122 (0.092–0.152)	0.518 (0.507–0.529)	0.088 (0.065–0.111)	0.948 (0.936–0.960)	0.380 (0.353–0.407)	0.346 (0.242–0.465)	0.769 (0.765–0.774)
	LR	0.609 (0.568–0.650)	0.374 (0.321–0.427)	0.567 (0.532–0.602)	0.460 (0.394–0.526)	0.674 (0.629–0.719)	0.466 (0.414–0.518)	0.361 (0.298–0.428)	0.758 (0.725–0.792)
	SVM (linear)	0.417 (0.364–0.470)	0.328 (0.275–0.381)	0.555 (0.526–0.585)	0.435 (0.359–0.511)	0.676 (0.621–0.731)	0.320 (0.276–0.364)	0.349 (0.275–0.432)	0.780 (0.754–0.806)
	SVM (radial)	0.391 (0.352–0.430)	0.264 (0.212–0.316)	0.533 (0.507–0.558)	0.405 (0.322–0.488)	0.660 (0.587–0.733)	0.297 (0.262–0.333)	0.323 (0.238–0.422)	0.752 (0.728–0.776)
ALC-PEER	RF	0.819 (0.801–0.836)	0.406 (0.368–0.443)	0.632 (0.615–0.650)	0.328 (0.292–0.364)	0.937 (0.925–0.949)	0.613 (0.580–0.647)	0.619 (0.549–0.689)	0.819 (0.811–0.827)
	LR	0.443 (0.405–0.481)	0.263 (0.217–0.309)	0.492 (0.464–0.521)	0.345 (0.282–0.408)	0.640 (0.591–0.689)	0.370 (0.329–0.411)	0.277 (0.216–0.344)	0.709 (0.670–0.744)
	SVM (linear)	0.393 (0.350–0.436)	0.308 (0.267–0.350)	0.477 (0.451–0.504)	0.515 (0.439–0.591)	0.440 (0.370–0.510)	0.328 (0.289–0.368)	0.269 (0.218–0.325)	0.661 (0.611–0.710)
	SVM (radial)	0.408 (0.370–0.446)	0.313 (0.268–0.358)	0.516 (0.493–0.540)	0.515 (0.436–0.594)	0.518 (0.441–0.595)	0.330 (0.292–0.368)	0.299 (0.238–0.370)	0.725 (0.700–0.751)
ALC-SOC	RF	0.719 (0.699–0.740)	0.257 (0.219–0.295)	0.562 (0.546–0.578)	0.210 (0.175–0.245)	0.914 (0.900–0.928)	0.446 (0.417–0.474)	0.432 (0.354–0.513)	0.789 (0.782–0.796)
	LR	0.695 (0.654–0.736)	0.477 (0.423–0.531)	0.642 (0.604–0.680)	0.570 (0.500–0.640)	0.714 (0.675–0.753)	0.549 (0.495–0.602)	0.444 (0.381–0.509)	0.818 (0.786–0.851)
	SVM (linear)	0.431 (0.377–0.485)	0.443 (0.398–0.489)	0.597 (0.567–0.627)	0.660 (0.588–0.732)	0.534 (0.461–0.607)	0.338 (0.295–0.382)	0.362 (0.304–0.427)	0.822 (0.792–0.852)
	SVM (radial)	0.308 (0.258–0.358)	0.360 (0.299–0.421)	0.592 (0.559–0.625)	0.540 (0.451–0.629)	0.644 (0.569–0.719)	0.265 (0.231–0.299)	0.378 (0.295–0.472)	0.813 (0.783–0.842)
ALC-NR	RF	0.739 (0.720–0.759)	0.204 (0.165–0.242)	0.543 (0.528–0.559)	0.162 (0.130–0.194)	0.924 (0.910–0.939)	0.456 (0.423–0.489)	0.401 (0.309–0.500)	0.780 (0.774–0.787)
	LR	0.482 (0.439–0.525)	0.333 (0.290–0.376)	0.510 (0.479–0.540)	0.465 (0.399–0.531)	0.554 (0.499–0.609)	0.407 (0.360–0.453)	0.294 (0.242–0.352)	0.721 (0.682–0.756)
	SVM (linear)	0.389 (0.347–0.431)	0.347 (0.305–0.390)	0.500 (0.473–0.527)	0.630 (0.548–0.712)	0.370 (0.296–0.444)	0.274 (0.242–0.305)	0.286 (0.237–0.339)	0.717 (0.669–0.765)
	SVM (radial)	0.404 (0.356–0.452)	0.287 (0.233–0.341)	0.539 (0.510–0.567)	0.445 (0.359–0.531)	0.632 (0.556–0.708)	0.325 (0.286–0.365)	0.326 (0.245–0.421)	0.763 (0.734–0.793)
PSY-PEER	RF	0.748 (0.728–0.769)	0.268 (0.223–0.314)	0.578 (0.559–0.597)	0.206 (0.168–0.244)	0.950 (0.939–0.961)	0.514 (0.482–0.546)	0.563 (0.460–0.664)	0.795 (0.786–0.803)
	LR	0.763 (0.741–0.784)	0.503 (0.468–0.538)	0.680 (0.656–0.704)	0.570 (0.522–0.618)	0.790 (0.769–0.811)	0.515 (0.480–0.550)	0.475 (0.429–0.522)	0.853 (0.839–0.868)
	SVM (linear)	0.749 (0.718–0.781)	0.506 (0.476–0.536)	0.677 (0.657–0.697)	0.552 (0.511–0.593)	0.801 (0.780–0.822)	0.538 (0.502–0.574)	0.481 (0.437–0.527)	0.848 (0.836–0.860)
	SVM (radial)	0.670 (0.633–0.708)	0.393 (0.352–0.435)	0.618 (0.596–0.640)	0.418 (0.369–0.467)	0.818 (0.792–0.844)	0.445 (0.411–0.478)	0.434 (0.372–0.499)	0.815 (0.802–0.827)
PSY-SOC	RF	0.598 (0.574–0.621)	0.135 (0.103–0.167)	0.502 (0.488–0.516)	0.118 (0.088–0.148)	0.886 (0.870–0.903)	0.321 (0.298–0.345)	0.245 (0.175–0.322)	0.763 (0.757–0.770)
	LR	0.638	0.385	0.578	0.476	0.681	0.397	0.332	0.798

Feature Category	Model	AUC	F1	Balanced Accuracy	Sensitivity	Specificity	PR-AUC	PPV	NPV
		(0.616–0.661)	(0.358–0.413)	(0.558–0.599)	(0.436–0.516)	(0.655–0.707)	(0.370–0.424)	(0.296–0.370)	(0.784–0.812)
	SVM (linear)	0.553 (0.521–0.586)	0.343 (0.307–0.379)	0.564 (0.542–0.587)	0.420 (0.369–0.471)	0.709 (0.684–0.733)	0.311 (0.286–0.336)	0.325 (0.280–0.370)	0.794 (0.779–0.809)
	SVM (radial)	0.396 (0.364–0.428)	0.129 (0.093–0.164)	0.517 (0.505–0.530)	0.162 (0.112–0.212)	0.873 (0.837–0.908)	0.237 (0.216–0.259)	0.298 (0.186–0.435)	0.764 (0.754–0.775)
PSY-NR	RF	0.569 (0.541–0.596)	0.132 (0.098–0.165)	0.510 (0.495–0.524)	0.102 (0.074–0.130)	0.917 (0.903–0.932)	0.327 (0.299–0.355)	0.279 (0.193–0.374)	0.766 (0.760–0.772)
	LR	0.620 (0.595–0.644)	0.325 (0.298–0.353)	0.566 (0.544–0.587)	0.465 (0.421–0.509)	0.667 (0.643–0.690)	0.337 (0.304–0.369)	0.259 (0.228–0.291)	0.835 (0.824–0.847)
	SVM (linear)	0.481 (0.449–0.513)	0.280 (0.248–0.312)	0.538 (0.515–0.562)	0.438 (0.381–0.494)	0.639 (0.609–0.669)	0.230 (0.208–0.252)	0.232 (0.196–0.272)	0.827 (0.813–0.841)
	SVM (radial)	0.399 (0.374–0.424)	0.122 (0.090–0.154)	0.488 (0.477–0.499)	0.305 (0.219–0.391)	0.671 (0.588–0.755)	0.181 (0.167–0.194)	0.188 (0.117–0.285)	0.792 (0.786–0.799)
PEER-SOC	RF	0.834 (0.816–0.851)	0.491 (0.454–0.528)	0.675 (0.656–0.695)	0.440 (0.399–0.481)	0.911 (0.897–0.924)	0.657 (0.626–0.689)	0.606 (0.548–0.664)	0.842 (0.832–0.852)
	LR	0.793 (0.771–0.814)	0.570 (0.541–0.598)	0.737 (0.714–0.759)	0.712 (0.667–0.757)	0.761 (0.742–0.781)	0.557 (0.520–0.594)	0.482 (0.447–0.519)	0.900 (0.886–0.915)
	SVM (linear)	0.790 (0.770–0.810)	0.555 (0.528–0.583)	0.724 (0.702–0.746)	0.704 (0.662–0.746)	0.744 (0.723–0.764)	0.552 (0.516–0.588)	0.462 (0.428–0.497)	0.895 (0.881–0.909)
	SVM (radial)	0.771 (0.746–0.795)	0.492 (0.457–0.527)	0.680 (0.658–0.702)	0.562 (0.514–0.610)	0.798 (0.777–0.819)	0.521 (0.487–0.555)	0.465 (0.419–0.513)	0.860 (0.847–0.873)
PEER-NR	RF	0.840 (0.818–0.861)	0.483 (0.440–0.525)	0.673 (0.652–0.693)	0.388 (0.347–0.429)	0.958 (0.948–0.967)	0.683 (0.647–0.719)	0.740 (0.674–0.804)	0.836 (0.827–0.846)
	LR	0.736 (0.714–0.758)	0.504 (0.479–0.528)	0.682 (0.662–0.701)	0.630 (0.592–0.668)	0.733 (0.713–0.753)	0.500 (0.469–0.531)	0.425 (0.392–0.458)	0.868 (0.855–0.880)
	SVM (linear)	0.686 (0.646–0.726)	0.501 (0.477–0.524)	0.678 (0.659–0.697)	0.632 (0.592–0.672)	0.723 (0.695–0.751)	0.499 (0.460–0.538)	0.416 (0.378–0.457)	0.869 (0.857–0.882)
	SVM (radial)	0.750 (0.720–0.780)	0.483 (0.451–0.514)	0.664 (0.643–0.684)	0.512 (0.470–0.554)	0.816 (0.785–0.847)	0.564 (0.527–0.600)	0.465 (0.405–0.530)	0.846 (0.835–0.857)
SOC-NR	RF	0.639 (0.615–0.663)	0.340 (0.304–0.376)	0.584 (0.565–0.603)	0.306 (0.270–0.342)	0.863 (0.841–0.884)	0.423 (0.392–0.454)	0.410 (0.346–0.480)	0.800 (0.791–0.809)
	LR	0.556 (0.535–0.576)	0.353 (0.331–0.376)	0.548 (0.529–0.567)	0.524 (0.482–0.566)	0.571 (0.544–0.599)	0.287 (0.267–0.306)	0.276 (0.248–0.306)	0.793 (0.764–0.819)
	SVM (linear)	0.483 (0.457–0.510)	0.378 (0.357–0.398)	0.555 (0.537–0.574)	0.666 (0.614–0.718)	0.445 (0.405–0.485)	0.257 (0.235–0.279)	0.273 (0.244–0.304)	0.851 (0.829–0.872)
	SVM (radial)	0.469 (0.439–0.498)	0.358 (0.337–0.380)	0.537 (0.524–0.551)	0.682 (0.614–0.750)	0.393 (0.316–0.469)	0.274 (0.247–0.300)	0.260 (0.219–0.306)	0.816 (0.796–0.835)

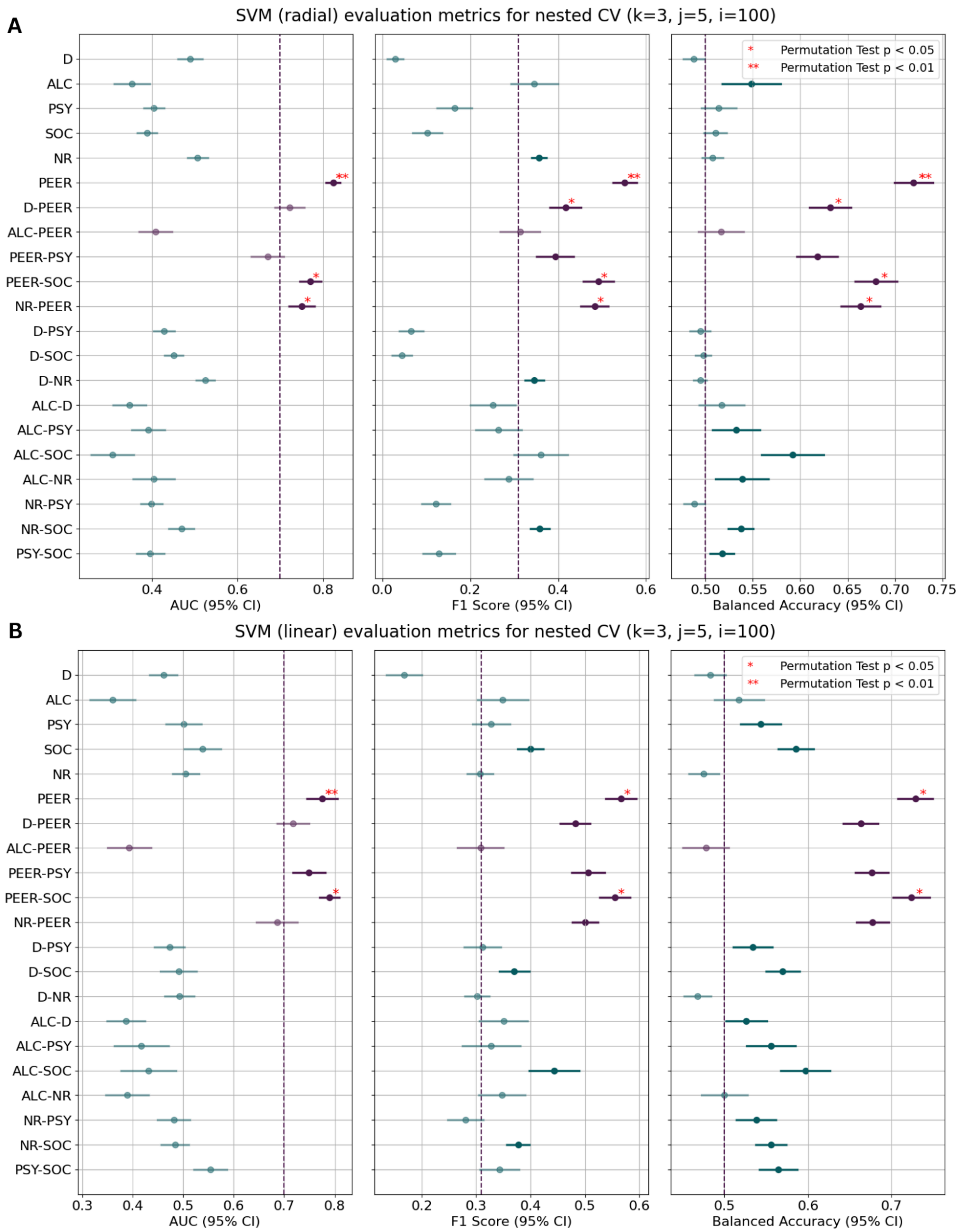

Figure S5. Predictive performance of SVM models trained using baseline alcohol use, psychological assessments, sociometric data, neural responses, peer drinking perceptions, and demographics, and their pairwise combinations, evaluated using nested cross-validation. Results are presented for (A) SVM with

radial basis function, and (B) linear SVM. For each model, performance metrics—including AUC (left panel), F1 Score (center), and Balanced Accuracy (right)—were averaged across 100 outer validation folds (30% holdout), using 3-fold outer and 5-fold inner cross-validation (k=3, j=5, i=100). Points represent mean performance, and error bars show 95% confidence intervals. Vertical dashed lines indicate the minimum thresholds for chance-level classification; due to class imbalance, the chance threshold for the F1 score is 0.31. Red asterisks indicate models that performed significantly better than chance based on permutation testing (p < .05 or p < .01); the p-values are corrected for multiple comparisons within feature domains across the selected metrics using Benjamini–Hochberg FDR correction. Predictor categories are abbreviated as follows: D = demographics, ALC = baseline alcohol use, PSY = psychological assessments, PEER = subjective peer drinking perceptions, SOC = sociometric nominations, and NR = neural responses to alcohol, social cues, and at rest.

External test results for all model types

Table S8. Evaluation of all model types (random forest, logistic regression using Elastic Net, linear SVM, and radial SVM) trained on PEER features on the unstratified out-of-sample test set. Performance metrics reflect model generalizability under natural class distribution in a completely external test set. Inference was run over the entire test set once, and metrics were computed accordingly. Hence, there are no confidence intervals or p-values to report for most metrics. For AUC, confidence intervals were calculated using the analytical DeLong method.

Metric	RF	LR (ElasticNet)	SVM (linear)	SVM (radial)
AUC	0.68<br>(0.54, 0.82)	0.67<br>(0.53, 0.83)	0.62<br>(0.48, 0.77)	0.73<br>(0.59, 0.87)
F1 score	0.40	0.31	0.30	0.32
Balanced accuracy	0.68	0.62	0.63	0.62
Sensitivity	0.50	0.43	0.50	0.36
Specificity	0.86	0.81	0.75	0.88
PR-AUC	0.68	0.29	0.27	0.33
PPV	0.33	0.24	0.22	0.29
NPV	0.92	0.91	0.91	0.91

For the 'PEER' features, RF model PDPs shown in Figure S6 illustrate how perceived peer behavior influences the prediction of intervention responsiveness in the test set from Study 2. For *perceived peer drinking amount*, a threshold effect similar to that observed during model validation (see Figure S1) emerges: once perceived consumption exceeds approximately three drinks per occasion, the likelihood of predicting a positive intervention response (shown on the y-axis) declines. Similar to the feature relationship during validation, *perceived peer drinking frequency* again exhibits a non-linear relationship with intervention effectiveness prediction: Participants who perceived their peers to drink on 60–80 occasions per year are most likely to receive a positive effectiveness prediction, whereas both lower and higher perceived peer drinking frequencies are associated with reduced likelihood of positive prediction. Thus, the

observed response dynamics closely mirror those identified in the validation sets, indicating consistent model behavior and robust predictor effects across data splits (Study 1 and Study 2).

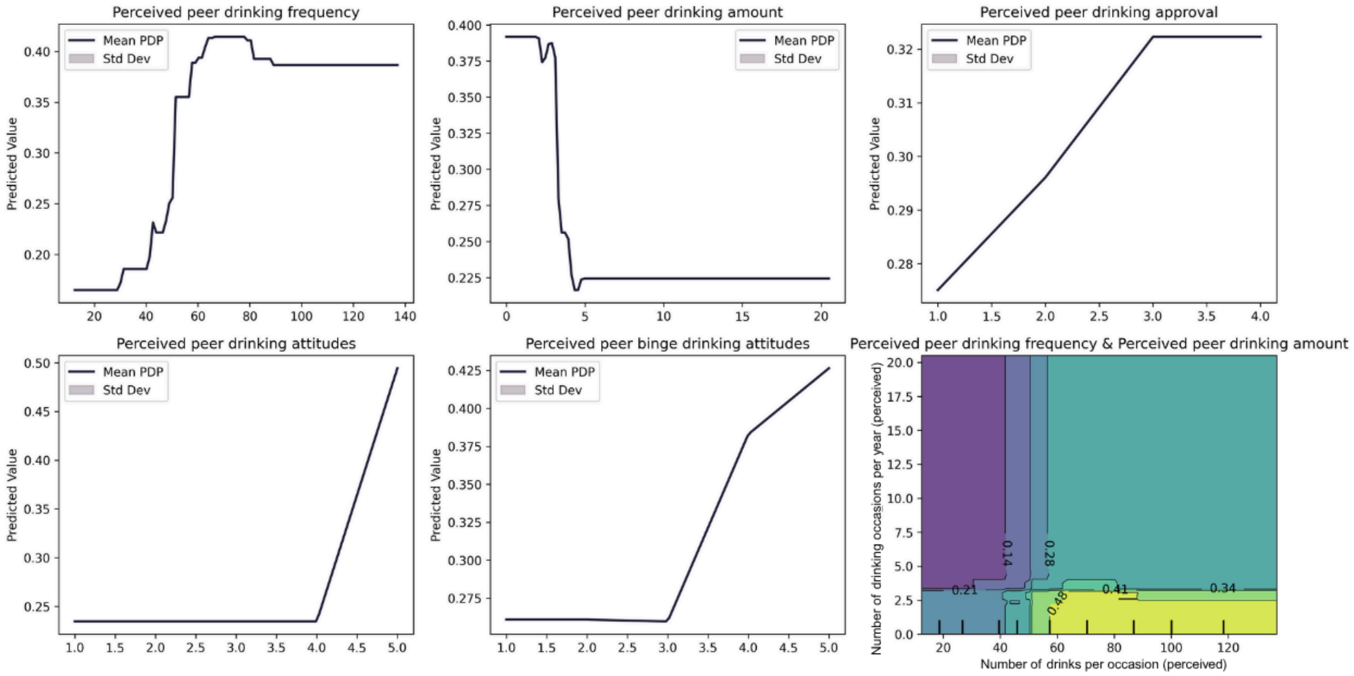

Figure S6. Partial dependence plots for key predictors based on the external test set (Study 2).

7 - Additional Sensitivity Analyses

Alternative CV Splits

Table S9. Performance metrics for the RF model sensitivity analyses using varying train/validation splits and cross-validation partitions. Results assess the robustness of model performance to different data partitioning strategies.

k	train/validation ratio	AUC *(95% CI)*	F1 *(95% CI)*	Balanced Accuracy *(95% CI)*
3	60/40	0.85 (0.84, 0.86)	0.51 (0.48, 0.55)	0.69 (0.67, 0.71)
5	70/30	0.83 (0.85, 0.88)	0.56 (0.53, 0.60)	0.71 (0.69, 0.73)

Alternative Responsiveness Thresholds

Table S10. Sensitivity analysis of RF model performance across thresholds for defining behavior change. Nested CV was performed with i=100, k=3, j=5. Performance is most balanced at the –1 average drinking occasions/week threshold. At –0.5, lower F1 and balanced accuracy suggest increased label noise from very small reductions in drinking occasions. Nonetheless, the model is able to retain above-chance prediction levels. At –2, the number of strong responders is very small, resulting in severe class imbalance. As a result, absolute performance metrics like F1 drop sharply despite an acceptable AUC.

Average change in the number of drinking occasions between active and control weeks	AUC *(95% CI)*	F1 *(95% CI)*	Balanced Accuracy *(95% CI)*
> -0.0	0.55 *(0.53, 0.57)*	0.50 *(0.47, 0.52)*	0.52 *(0.51, 0.54)*
> -0.5	0.72 *(0.70, 0.74)*	0.46 *(0.43, 0.49)*	0.64 *(0.62, 0.65)*
> -1.0	**0.87** ***(0.85–0.88)***	**0.55** ***(0.51–0.59)***	**0.71** ***(0.69–0.73)***
> -2.0	0.77 *(0.74, 0.79)*	0.13 *(0.08, 0.17)*	0.53 *(0.51, 0.56)*

Drinking on Control Weeks

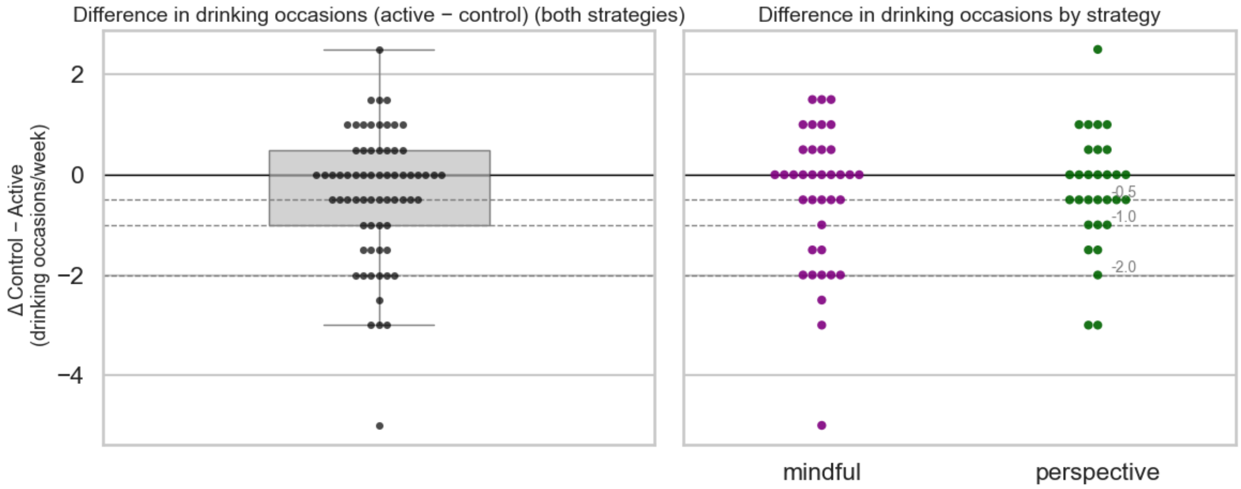

Figure S7. Difference in the average number of drinking occasions between active and control weeks. The left panel shows the difference for all intervention participants. The right panel shows the change for both intervention strategies separately. Thresholds -0.5, -1.0, and -2.0 are indicated in the figure. Negative values reflect that a participant reduced their drinking in active weeks compared to control weeks.

Negative Control (Placebo) Analysis

Table S11. Performance metrics from the placebo analysis using control-only participants assigned pseudo-intervention sequences (on–off–on–off vs. off–on–off–on). Models were trained on the PEER feature group using 3-fold cross-validation, 100 repetitions, and a 70/30 train-validation split. Results indicate no above-chance predictability, supporting the intervention-driven nature of effects.

**Condition**	**AUC** *(95% CI)*	**F1** *(95% CI)*	**Balanced Accuracy** *(95% CI)*
on-off-on-off	0.67 *(0.64, 0.59)*	0.24 *(0.20, 0.28)*	0.55 *(0.53, 0.57)*
off-on-off-on	0.61 *(0.58, 0.63)*	0.34 *(0.31, 0.38)*	0.56 *(0.54, 0.58)*

Baseline Drinking Levels

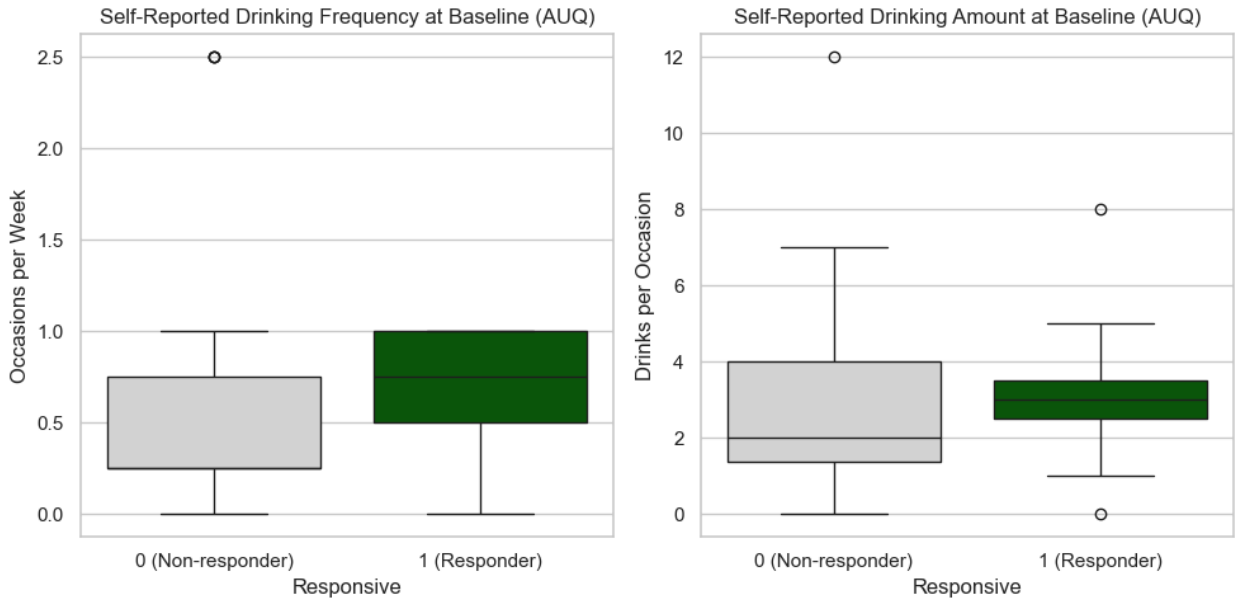

Figure S8. Self-reported drinking frequency (in occasions per week) and drinking amount (in number of drinks per drinking occasion) for responders and non-responders in Study 1 at baseline. We observed no significant differences (Welch's t-test) in baseline drinking amount or frequency between individuals who were responsive and those who were non-responsive to the psychological distancing interventions (drinking frequency: $t(18.10) = 1.62$, $p = 0.122$; drinking amount: $t(17.67) = 0.53$, $p = 0.603$).

Drinking on Control vs Active Weeks

Table S12. Drinking behavior in terms of drinking frequency and drinking amount per occasion during the intervention time by study and responsiveness status. Participants in the control group were included for comparison.

	Study 1			Study 2	
**Intervention time drinking**	**Responders (n=15)**	**Non-Responders (n=52)**	**Controls (n=37)**	**Responders (n=14)**	**Non-Responders (n=100)**
drinking **amount** on **control** weeks (drinks/occasion), M, SD, (Min-Max)	2.95, 0.98, (1.5-4.83)	3.13, 1.79, (1.0-7.5)	3.6, 1.86, (0.0-8.67)	1.77, 0.59, (1.0-8.0)	2.10, 1.37, (1.0-8.0)
drinking **amount** on **active** weeks (drinks/occasion), M, SD, (Min-Max)	2.14, 0.90, (1.0-4.0)	3.07, 1.82, (1.0-8.0)	-	2.11, 2.02, (1.0-8.0)	2.25, 1.2, (1.0-6.67)
drinking **frequency** on **control** weeks (drinking occasions/week), M, SD, (Min-Max)	4.0, 1.93, (2.5-9.5)	1.68, 1.15, (0.5-5.5)	2.2, 1.59, (0.0-6.25)	3.45, 1.04, (2.0-5.0)	1.76, 1.09, (0.5-5.0)
drinking **frequency** on **active** weeks (drinking occasions/week), M, SD, (Min-Max)	1.73, 1.32, (0.5-4.5)	1.87, 1.13, (0.5-5.0)	-	1.41, 0.7, (0.5-2.5)	1.86, 1.42, (0.5-7.0)

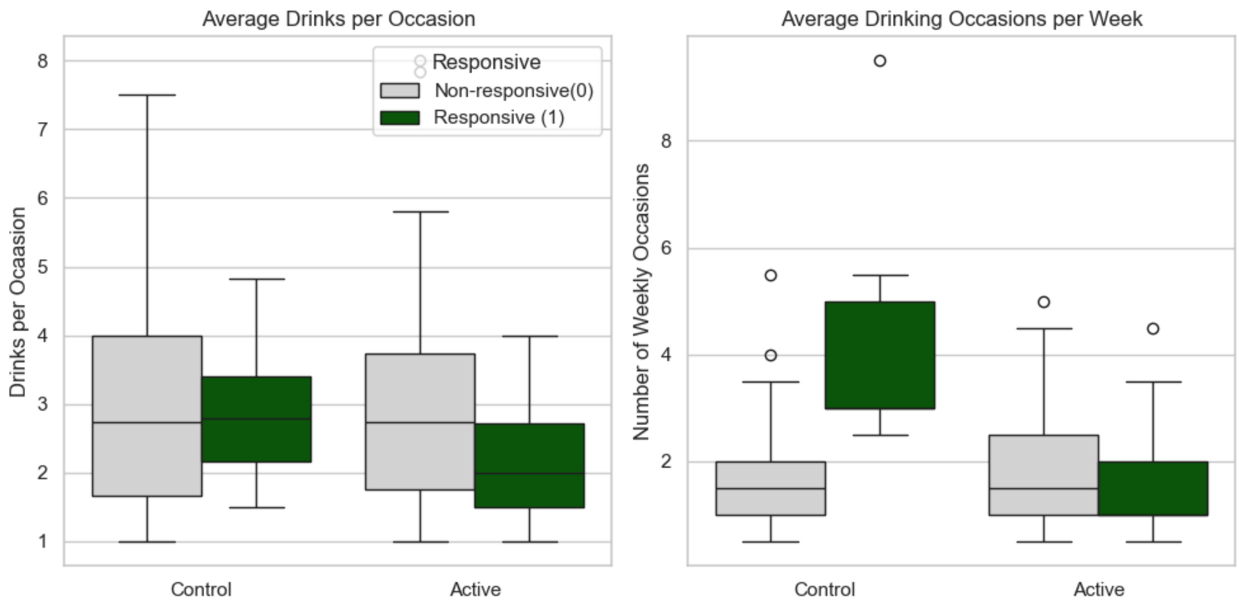

Figure S9. Drinking frequency and drinking amount during the Study 1 intervention period. The x-axis shows control weeks and active weeks, with responsive individuals shown in green and non-responsive individuals shown in grey. In terms of drinking amount, individuals do not differ strongly by responder status. However, in control weeks, responsive individuals appeared to be more frequent drinkers than non-responders. This includes only individuals assigned to the intervention (no controls).

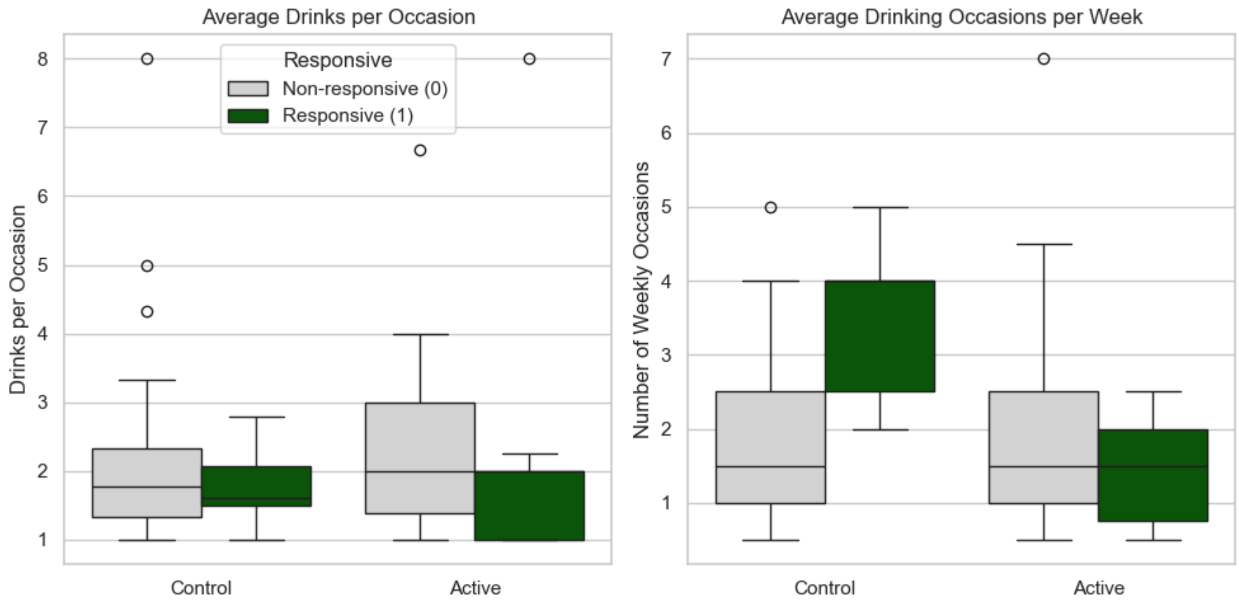

Figure S10. Drinking frequency and drinking amount during the Study 2 intervention period. The x-axis shows control weeks and active weeks, with responsive individuals shown in green and non-responsive individuals shown in grey. In terms of drinking amount, individuals do not differ strongly by responder status. However, in control weeks, responsive individuals appeared to be more frequent drinkers than non-responders. This includes only individuals assigned to the intervention (no controls).

Table S13. **Comparison of drinking frequency and amount between responders and non-responders during control and active intervention weeks.** T-tests were used to assess group differences. Significant differences ($p < .05$) indicate that responders drank more frequently during control weeks than non-responsive individuals in both studies and consumed fewer drinks per occasion during active intervention weeks compared to non-responsive individuals in Study 1.

	Study 1		Study 2	
Measure	t-statistic	p-value	t-statistic	p-value
Control – Drinks per Occasion	-0.455	0.652	-1.136	0.263
Active – Drinks per Occasion	-2.392	0.021	-0.219	0.83
Control – Drinking Occasions per Week	4.092	0.001	4.677	<0.001
Active – Drinking Occasions per Week	-0.337	0.74	-1.424	0.163

Accuracy of Peer Perceptions

To assess the accuracy of peer perceptions, we compared each participant's perceived drinking behavior of their nominated peers to the "true" (i.e., self-reported) behavior of those peers. For drinking amount, perceptions were close to the true average at the group level ($M_{self\text{-}report} = 4.89$, $M_{perceived} = 4.52$ drinks per drinking occasion), but individual-level agreement was low (Spearman $\rho = 0.34$), as shown by wide variability in the scatterplot (Figure S3A). For drinking frequency, peers substantially underestimated "true" (i.e., self-reported) behavior ($M_{self\text{-}report} = 1.9$, $M_{perceived} = 1.06$), with even weaker individual-level agreement (Spearman $\rho = 0.25$); see Table S10. Both scatterplots reveal large individual discrepancies and suggest that peer estimates are inconsistent and inaccurate when evaluated against actual self-reports.

Complete data for this analysis were available for 46 participants in Study 1. Some participants nominated peers and reported their perceptions, but those peers did not complete self-reports on baseline drinking, reducing the available matched data. Similarly, as self-reported baseline drinking data were not generally available for all Study 2 participants, the analysis could only be performed for 40 individuals from Study 2.

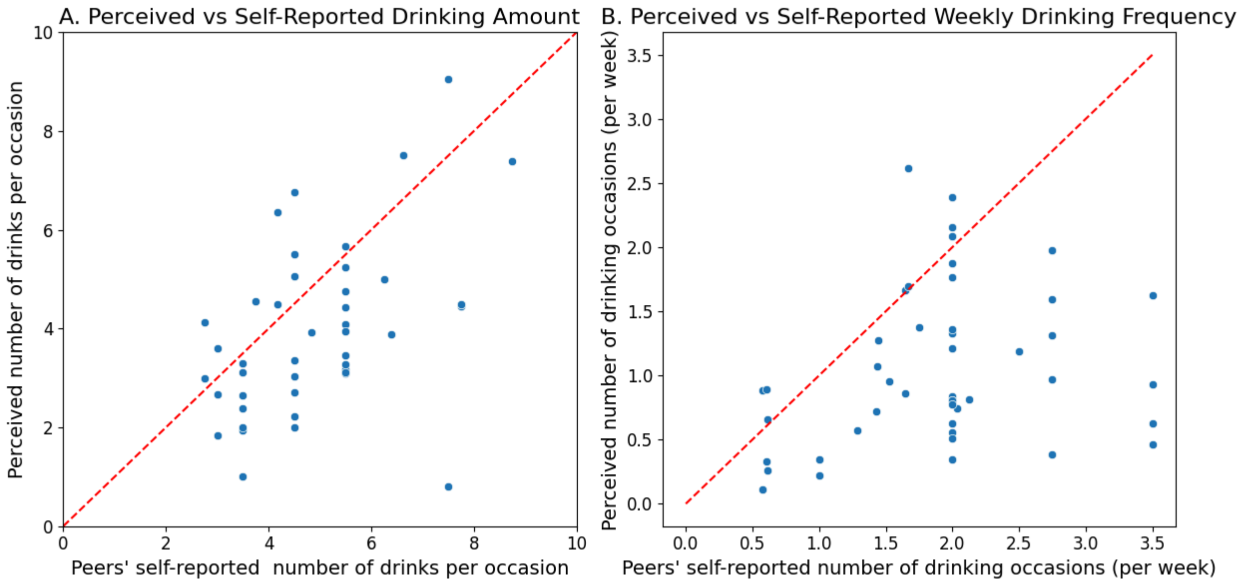

Figure S11. Scatterplots comparing the average of perceived peer drinking in individuals' "highest drinker" nominations versus the average of those peers' self-reports of their drinking behavior in Study 1. Each point represents one participant's perception of their nominated peers' average (A) drinking amount and (B) drinking frequency, plotted against the average self-reported values from those peers. The red dashed line indicates perfect agreement (y = x). While perceived drinking amount is approximately accurate on average, perceptions of drinking frequency show a systematic underestimation of peers' drinking frequencies.

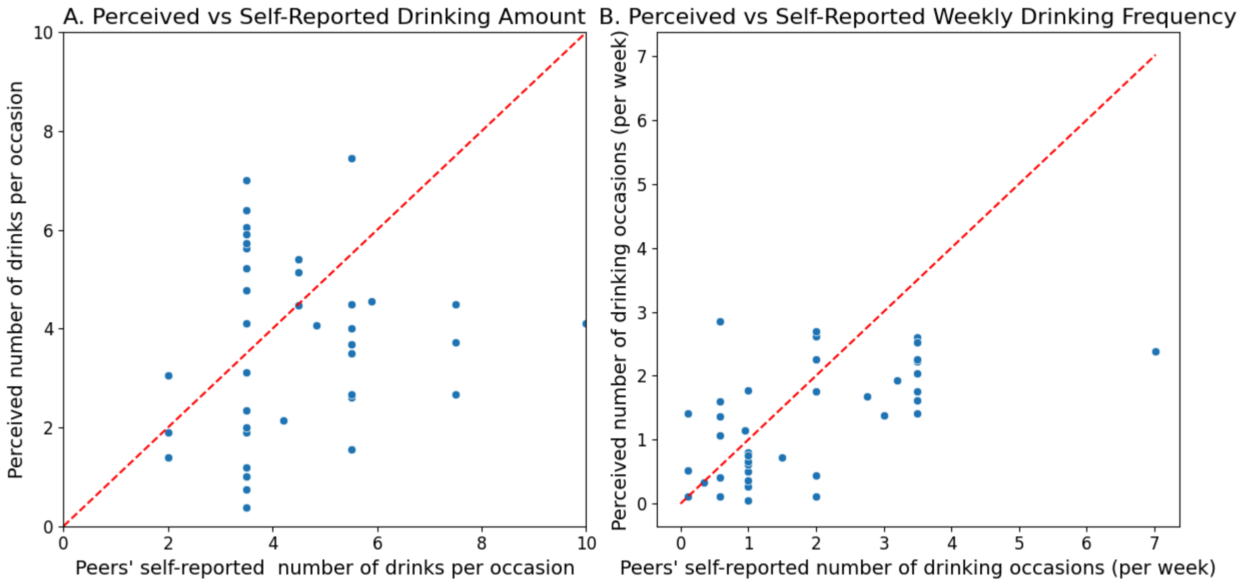

Figure S12. Scatterplots comparing the average of perceived peer drinking in individuals' "highest drinker" nominations versus the average of those peers' self-reports of their drinking behavior in Study 2. Each point represents one participant's perception of their nominated peers' average (A) drinking amount and (B) drinking frequency, plotted against the average self-reported values from those peers. The red dashed line indicates perfect agreement (y = x). Perceptions of drinking frequency show a slight underestimation of peers' drinking frequencies.

Table S14. Mean and standard deviations of the self-reported and perceived peer drinking amount and behavior. The mean absolute error (MAE) between the perceptions and self-reports, as well as correlation coefficients (Pearson and Spearman), are reported.

	**Study 1**				**Study 2**			
	**Amount (drinks per occasion)**		**Frequency (drinks per week)**		**Amount (drinks per occasion)**		**Frequency (drinks per week)**	
**Metric**	self-report	peer perception	self-report	peer perception	self-report	peer perception	self-report	peer perception
M, SD	4.89, 1.56	4.52, 3.31	1.9, 0.78	1.06, 0.61	4.44, 1.64	3.80, 2.12	1.84, 1.44	1.35, 0.88
Mean Absolute Error (MAE)	2.14		0.95		2.17		0.97	
Pearson r	-0.01		0.25		0.08		0.57	
Wilcoxon signed-rank test (p-value)	0.003		<0.001		0.087		0.013	

Table S15. Statistical significance tests investigating whether participants who underestimate their peers' drinking frequency and amount are possibly less socially integrated in their peer groups. We used Mann-Whitney U tests to compare individuals' self-reported drinking frequency and quantity across different levels of sociometric in-degree (positions in their social networks).We found no statistically significant differences between participants who underestimate and those who do not, suggesting that underestimation is not driven by atypical drinking patterns or lower social integration.

**Feature**	**p-value (t-test)**	**p-value (Mann-Whitney U)**
AUQ_drink_intent	0.125	0.296
AUQ_drink_frequency	0.948	0.841
AUQ_drink_amount	0.661	0.640
In-degree of "liked" peer network (*like_deg_in*)	0.709	0.661
In-degree of "least drinkers" peer network (*alcLeast_deg_in)*	0.205	0.149
In-degree of "highest drinkers" network *(alcMost_deg_in)*	0.743	0.857
In-degree of "closest" peer network *(closest_deg_in)*	0.537	0.354

Table S16. Full set of evaluation metrics of the RF model trained on actual drinking (not drinking perceptions) of an individual's social group. Features used for prediction were: ID of the group the individual was a member of, average drinking frequency (drinking occasions per year) of their social group members (taken from those members' self-reports), and average drinking amount (number of drinks per occasion) of their social group members (taken from those members' self-reports). The table shows the results of the 100 runs in the cross-validation process on Study 1 data. The cross-validation process was the same as for the model development described in the main manuscript.

Metric	Value (95% CI)
AUC	0.48 (0.46, 0.50)
F1 score	0.29 (0.27, 0.33)
Balanced accuracy	0.54 (0.52, 0.56)
Sensitivity	0.34 (0.30, 0.34)
Specitivity	0.74 (0.72, 0.77)
PR-AUC	0.30 (0.27, 0.32)
PPV	0.30 (0.26, 0.33)
NPV	0.78 (0.77, 0.79)

Table S17. Test-retest stability of self-reports on drinking frequency and amount, peer perceptions of drinking frequency and amount, and peer nominations for heaviest drinkers. The Jaccard index measures how many of the same peers were nominated at both time points, calculated as the number of overlapping nominations divided by the total number of unique nominations across waves. Spearman's rank correlation coefficient measures how consistently participants' scores ranked between baseline and follow-up (after six months).

Comparison of baseline vs 6-month follow-up	Spearman coefficient ρ
self-reports number of drinking occasions	$\rho = 0.54$, $p < 0.001$
self-reports number of drinks per occasion	$\rho = 0.46$, $p < 0.001$
peer-perceptions average number of drinking occasions of nominated peers	$\rho = 0.47$, $p = 0.007$
peer-perceptions average number of drinks per occasions of nominated peers	$\rho = 0.20$, $p = 0.279$
	Jaccard Index
nominations of heaviest drinking peers (*alcMost*)	0.267

Mediation analyses

We conducted follow-up mediation analyses to explore potential mechanisms of why the interventions may be more (vs. less) effective for participants who perceived moderate levels of peer drinking. Drawing on social norms literature[53,54], when peers are perceived to drink in moderation and pro-drinking approval is moderate—salient enough to matter, but not extreme, psychological distancing interventions may have the most room to reduce the influence of social alcohol cues on behavior. For example, if peers rarely drink or disapprove of drinking, there may be little relevance for the intervention to target; conversely, if peers drink heavily and strongly approve of drinking, intervention impact may be constrained by a ceiling effect. Thus, we tested whether perceived drinking approval helps explain (i.e., mediate) the relationship between perceived peer drinking frequency and intervention effectiveness. These exploratory analyses allow us to assess whether changes in perceived group drinking approval may be one plausible pathway through which the intervention exerts its effects.

In Study 1 (n=67), results indicate a marginally significant average causal mediation effect (ACME), suggesting that the indirect effect through perceived approval was small (ACME = 0.023, 95% CI [–0.001, 0.066], p = 0.062). The average direct effect (ADE) of perceived peer drinking on intervention effectiveness, independent of the mediator, was significant (ADE = 0.230, 95% CI [0.116, 0.345], p < 0.001). The total effect combining direct and indirect pathways was also significant (Total Effect = 0.254, 95% CI [0.144, 0.362], p < 0.001). The proportion of the total effect mediated was modest at 9.2% (95% CI [–0.003, 0.301], p = 0.062), meaning that about 9% of the total effect of perceived peer drinking on intervention effectiveness (responsive vs. non-responsive as a binary outcome) was explained via the mediator (perceived group drinking approval). In Study 2 (*N*=114), the mediation analysis showed that perceived group approval partially mediated the effect of alcohol frequency on the outcome. Specifically, the indirect effect via the mediator was significant (ACME β = 0.049, 95% CI [0.018, 0.092], p < 0.001), while the direct effect remained significant (ADE β = 0.102, 95% CI [0.038, 0.161], *p* = 0.002). The total effect was also significant (β = 0.151, 95% CI [0.096, 0.207], p < 0.001), with approximately 33% of the effect mediated (95% CI [0.124, 0.672], p < 0.001) (Figure S16). These findings provide only preliminary hypothesis-generating evidence that perceived group approval may partially explain the relationship between perceived alcohol consumption frequency of peers and intervention outcomes. However, further work is needed to prospectively test these hypotheses. Future work may explicitly test this mechanism using experimental designs that manipulate perceived group approval. For instance, participants could be randomly assigned to receive feedback indicating high or low peer approval of drinking at the start of the intervention, followed by prospective measurement of drinking outcomes. This approach would allow a stronger test of whether altering perceived approval influences individual differences in intervention effectiveness.

A Study 1 Mediation path diagram (partial mediation)

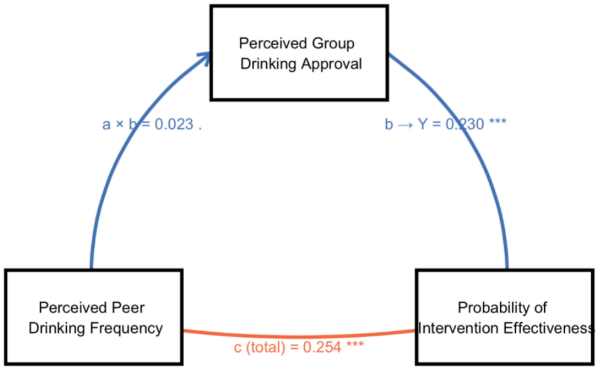

B Study 2 Mediation path diagram (partial mediation)

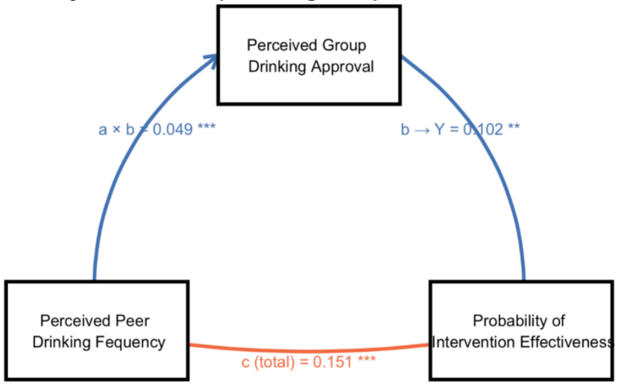

Figure S16. Mediation path diagrams for Study 1 (panel A) and Study 2 (panel B).

Model-free evidence

Table S18. Results of non-parametric significance tests comparing responders and non-responders across all feature domains and variables. For each variable, either a Mann–Whitney U test (continuous variables), Fisher's exact test (binary categorical variables), or Chi-square test (categorical variables with >2 levels) was conducted to assess group differences. Means and standard deviations for the responder and the non-responder groups are reported to assess the direction of differences, and p-values were corrected for multiple comparisons within each domain using the Benjamini–Hochberg False Discovery Rate (FDR) procedure. The table reports the domain, variable name, test statistic, mean/standard deviation, and FDR-corrected p-value for each variable. Statistically significant differences (*p<0.05*) are marked with *.

domain	variable	test statistic	Non-Responders (M, ±SD)	Responders (M, ±SD)	p (FDR)
ALC	AUQ_drink_frequency	276.5	1.38 (±1.21)	1.94 (±0.93)	0.176
	alcohol_alc_att_2	573.5	4.82 (±0.70)	4.31 (±0.67)	0.176
	DEQ_reduced_cognition_exp	250.5	2.93 (±0.73)	3.37 (±0.61)	0.176
	DEQ_increased_confidence_exp	268.0	3.76 (±0.60)	4.10 (±0.56)	0.265
	alc_intent_binge	318.0	2.43 (±1.05)	2.88 (±0.81)	0.302
	DEQ_increased_sexual_exp	284.5	3.31 (±1.26)	3.93 (±0.96)	0.302
	DMQ_drinking_enhancement_motive	323.0	2.59 (±0.85)	2.99 (±1.00)	0.347
	alc_binge_efficacy	336.0	2.15 (±0.99)	2.50 (±1.03)	0.380
	alcohol_alc_att_1	346.0	5.41 (±0.87)	5.56 (±0.55)	0.476
	DEQ_reduced_tension_exp	324.0	2.82 (±0.75)	3.10 (±1.04)	0.516
	AUQ_drink_amount	363.0	2.97 (±2.43)	3.12 (±1.82)	0.558
	DMQ_drinking_coping_motive	419.0	1.94 (±0.83)	2.06 (±1.06)	0.989
	DMQ_drinking_social_motive	406.0	3.28 (±0.93)	3.35 (±0.84)	0.989
	DMQ_drinking_conformity_motive	422.5	1.75 (±0.69)	1.81 (±0.83)	0.989
	AUQ_drink_intent	434.0	2.45 (±1.07)	2.38 (±1.50)	0.989
	DEQ_negative_consequences_exp	383.0	2.35 (±0.54)	2.36 (±0.47)	0.989
BR	segregation_resting_state_total	541.0	0.59 (±0.08)	0.57 (±0.03)	0.138
	ROI_alc_react_v_rest_neurosynth_reward	460.0	0.33 (±0.94)	0.17 (±0.81)	0.718
	ROI_alc_react_v_rest_VS	434.0	0.05 (±1.22)	-0.14 (±0.98)	0.793
	mentalizing	387.0	0.14 (±0.33)	0.12 (±0.35)	0.882
D	college_year	332.0	-	-	0.447
	gender_numeric	0.4	-	-	0.447
	age	389.5	20.45 (±1.81)	20.56 (±1.50)	0.777
	race_numeric	460.5	-	-	0.777
	income_numeric	363.0	133213.96 (±62900.99)	134666.33 (±65723.26)	0.948
SOC	goToGood_deg_in	572.5	2.91 (±1.67)	1.88 (±1.67)	0.258
	alcLeast_deg_in	349.5	2.49 (±4.36)	2.50 (±2.83)	0.371
	alcMost_deg_in	531.5	5.26 (±6.62)	2.06 (±2.74)	0.371
	closest_deg_in	523.5	5.49 (±3.26)	4.44 (±3.41)	0.371
	influence_deg_in	506.5	3.09 (±5.82)	0.81 (±1.47)	0.371
	leaders_deg_in	494.0	4.15 (±7.38)	1.12 (±2.06)	0.371

domain	variable	test statistic	Non-Responders (M, ±SD)	Responders (M, ±SD)	p (FDR)
	goToBad_deg_in	489.0	2.38 (±1.89)	1.88 (±1.82)	0.402
	like_deg_in	460.0	7.72 (±4.30)	6.69 (±3.50)	0.612
PEER	avg_alcmost *	630.0	4.53 (±3.18)	2.73 (±1.16)	0.014
	groupAtt_alc *	243.5	4.08 (±0.81)	4.56 (±1.03)	0.014
	avg_alcmost_freq *	262.5	53.86 (±33.98)	70.10 (±25.45)	0.035
	alc_norm_5_r *	276.0	1.94 (±0.91)	2.62 (±1.15)	0.035
	groupAtt_binge *	282.0	3.09 (±1.11)	3.75 (±1.24)	0.038
PSY	DERS_goals	594.5	3.73 (±0.95)	2.92 (±1.11)	0.157
	DERS_mean	553.5	2.60 (±0.68)	2.24 (±0.46)	0.403
	DERS_clarity	503.0	2.48 (±0.81)	2.17 (±0.60)	0.590
	DERS_impulse	501.5	1.81 (±0.86)	1.54 (±0.74)	0.590
	DERS_nonacceptance	507.0	2.78 (±1.12)	2.25 (±0.79)	0.590
	BIS_motor_total	324.0	1.89 (±0.32)	2.08 (±0.48)	0.590
	STAI_mean	399.5	2.03 (±0.43)	2.01 (±0.39)	0.941
	ACS_attentionshifting	356.5	2.45 (±0.42)	2.54 (±0.55)	0.941
	ACS_mean	371.5	2.54 (±0.41)	2.64 (±0.45)	0.941
	CESD_mean	402.5	1.09 (±0.47)	1.08 (±0.48)	0.941
	MAAS_mean	426.5	3.49 (±0.83)	3.49 (±0.57)	0.941
	RPI_mean	429.0	2.66 (±0.38)	2.65 (±0.35)	0.941
	IAS_mean	347.0	2.85 (±1.05)	3.00 (±1.10)	0.941
	PILS_mean	407.0	4.25 (±0.70)	4.35 (±0.59)	0.941
	ULS.4_mean	452.5	1.53 (±0.58)	1.44 (±0.59)	0.941
	BIS_mean	364.0	2.07 (±0.29)	2.18 (±0.40)	0.941
	BIS_nonplanning	386.5	2.14 (±0.36)	2.18 (±0.45)	0.941

Citation statement

We sought to proactively consider choosing references that reflect the diversity of the field in terms of thought, form of contribution, and gender. First, we obtained the predicted gender of the first and last author of each reference by using databases that store the probability of a first name being carried by a woman (using https://github.com/dalejn/cleanBib). By this measure and excluding self-citations to the first and last authors of our current paper, our references contain 26% woman(first)/woman(last), 14% man/woman, 21% woman/man, and 39% man/man. This method is limited in that a) names, pronouns, and social media profiles used to construct the databases may not, in every case, be indicative of gender identity and b) it cannot account for intersex, non-binary, or transgender people. We look forward to future work that could help us to better understand how to support equitable practices in science.

References

1. Benitez, B. & Goldman, M. S. Using future-oriented expectancy associates to probe real-time variations in motivation to consume alcohol. *Psychol. Addict. Behav. J. Soc. Psychol. Addict. Behav.* **33**, 540–551 (2019).
2. Tyler, K. A., Schmitz, R. M., Adams, S. A. & Simons, L. G. Social factors, alcohol expectancy, and drinking behavior: A comparison of two college campuses. *J. Subst. Use* **22**, 357–364 (2017).
3. Burden, J. L. & Maisto, S. A. Expectancies, evaluations and attitudes: prediction of college student drinking behavior. *J. Stud. Alcohol* **61**, 323–331 (2000).
4. Wardell, J. D. & Read, J. P. Alcohol Expectancies, Perceived Norms and Drinking Behavior among College Students: Examining the Reciprocal Determinism Hypothesis. *Psychol. Addict. Behav. J. Soc. Psychol. Addict. Behav.* **27**, 191–196 (2013).
5. Linden, A. N., Lau-Barraco, C. & Milletich, R. J. Protective behavioral strategies, alcohol expectancies, and drinking motives in a model of college student drinking. *Psychol. Addict. Behav. J. Soc. Psychol. Addict. Behav.* **28**, 952–959 (2014).
6. Beard, S. J., Yoon, L., Venticinque, J. S., Shepherd, N. E. & Guyer, A. E. The brain in social context: A systematic review of substance use and social processing from adolescence to young adulthood. *Dev. Cogn. Neurosci.* **57**, 101147 (2022).
7. Jovanova, M. *et al.* Brain system integration and message consistent health behavior change. *Health Psychol. Off. J. Div. Health Psychol. Am. Psychol. Assoc.* **41**, 611–620 (2022).
8. Morgenstern, J., Naqvi, N. H., Debellis, R. & Breiter, H. C. The contributions of cognitive neuroscience and neuroimaging to understanding mechanisms of behavior change in addiction. *Psychol. Addict. Behav. J. Soc. Psychol. Addict. Behav.* **27**, 336–350 (2013).

9. Tanabe, J., Regner, M., Sakai, J., Martinez, D. & Gowin, J. Neuroimaging reward, craving, learning, and cognitive control in substance use disorders: review and implications for treatment. *Br. J. Radiol.* **92**, 20180942 (2019).

10. Hochster, A., Block-Lerner, J., Marks, D. R. & Erblich, J. Mindfulness buffers the effects of cue-induced craving on alcohol demand in college drinkers. *Addict. Behav.* **84**, 53–56 (2018).

11. Kirk-Provencher, K. T. *et al.* Neural responses to reward, threat, and emotion regulation and transition to hazardous alcohol use. *Alcohol Alcohol. Oxf. Oxfs.* **59**, agae043 (2024).

12. Naqvi, N. H. *et al.* Cognitive Regulation of Craving in Alcohol Dependent and Social Drinkers. *Alcohol. Clin. Exp. Res.* **39**, 343–349 (2015).

13. Lannoy, S. *et al.* Disentangling the role of social cognition processes at early steps of alcohol abuse: The influence of affective theory of mind. *Addict. Behav.* **102**, 106187 (2020).

14. Jovanova, M. *et al.* Neural responses to peers moderate conversation–drinking associations in daily life. *Sci. Rep.* (2025).

15. Staff, T. P. O. Correction: Modular Brain Network Organization Predicts Response to Cognitive Training in Older Adults. *PLOS ONE* **12**, e0174570 (2017).

16. Chen, Q. *et al.* Increased segregation of structural brain networks underpins enhanced broad cognitive abilities of cognitive training. *Hum. Brain Mapp.* **42**, 3202–3215 (2021).

17. Caldwell, T. M. *et al.* Patterns of association between alcohol consumption and symptoms of depression and anxiety in young adults. *Addict. Abingdon Engl.* **97**, 583–594 (2002).

18. Dvorak, R. D. *et al.* Alcohol use and alcohol-related consequences: associations with emotion regulation difficulties. *Am. J. Drug Alcohol Abuse* **40**, 125–130 (2014).

19. Willem, L., Vasey, M. W., Beckers, T., Claes, L. & Bijttebier, P. Cognitive biases and alcohol use in adolescence and young adulthood: The moderating role of gender, attentional control and inhibitory control. *Personal. Individ. Differ.* **54**, (2013).

20. Lyvers, M., Makin, C., Toms, E., Thorberg, F. A. & Samios, C. Trait mindfulness in relation to emotional self-regulation and executive function. *Mindfulness* **5**, 619–625 (2014).
21. Brett, E. I., Leffingwell, T. R. & Leavens, E. L. Trait mindfulness and protective strategies for alcohol use: Implications for college student drinking. *Addict. Behav.* **73**, 16–21 (2017).
22. Kang, Y. *et al.* Purpose in life, neural alcohol cue reactivity and daily alcohol use in social drinkers. *Addict. Abingdon Engl.* **117**, 3049–3057 (2022).
23. Crawford, L. A. & Novak, K. B. Resisting peer pressure: Characteristics associated with other-self discrepancies in college students' levels of alcohol consumption. *J. Alcohol Drug Educ.* **51**, 35–62 (2007).
24. Wootton, R. E. *et al.* Bidirectional effects between loneliness, smoking and alcohol use: evidence from a Mendelian randomization study. *Addict. Abingdon Engl.* **116**, 400–406 (2021).
25. Valente, T. W. *Social Networks and Health: Models, Methods, and Applications*. (Oxford University Press, New York, 2010).
26. Cole, V. T. *et al.* Peer connectedness and substance use in adolescence: A systematic review and meta-analysis. *Psychol. Addict. Behav. J. Soc. Psychol. Addict. Behav.* **38**, 19–35 (2024).
27. Bullers, S., Cooper, M. L. & Russell, M. Social network drinking and adult alcohol involvement: A longitudinal exploration of the direction of influence. *Addict. Behav.* **26**, 181–199 (2001).
28. Farrow, S. A Closer Look at the Impact of Peer Influences on College Drinking: Testing Moderation of Normative Drinking Perceptions by Social Network Characteristics. (University Name Unknown, 2009).
29. Borsari, B. & Carey, K. B. Peer influences on college drinking: a review of the research. *J. Subst. Abuse* **13**, 391–424 (2001).

30. Boyle, H. K., Merrill, J. E. & Carey, K. B. Location-Specific Social Norms and Personal Approval of Alcohol Use are Associated with Drinking Behaviors in College Students. *Subst. Use Misuse* **55**, 1650–1659 (2020).

31. Reid, A. E. & Carey, K. B. Why is social network drinking associated with college students' alcohol use? Focus on psychological mediators. *Psychol. Addict. Behav. J. Soc. Psychol. Addict. Behav.* **32**, 456–465 (2018).

32. Smith, P. C., Schmidt, S. M., Allensworth-Davies, D. & Saitz, R. Primary Care Validation of a Single-Question Alcohol Screening Test. *J. Gen. Intern. Med.* **24**, 783–788 (2009).

33. Townshend, J. M. & Duka, T. Patterns of alcohol drinking in a population of young social drinkers: a comparison of questionnaire and diary measures. *Alcohol Alcohol. Oxf. Oxfs.* **37**, 187–192 (2002).

34. National Epidemiologic Survey on Alcohol and Related Conditions (NESARC) - III. National Institutes of Health (NIH), Department of Health & Human Services (2023).

35. Cooper, M. L. Motivations for alcohol use among adolescents: Development and validation of a four-factor model. *Psychol. Assess.* **6**, 117–128 (1994).

36. Patton, K. A. *et al.* Validation of the Adolescent Drinking Expectancy Questionnaire and development of a short form. *Drug Alcohol Rev.* **37**, 396–405 (2018).

37. Spielberger, C. D., Gorsuch, R. L., Lushene, R., Vagg, P. R. & Jacobs, G. A. *Manual for the State-Trait Anxiety Inventory (Form Y)*. (Consulting Psychologists Press, Palo Alto, CA, 1983).

38. Radloff, L. S. The CES-D Scale: A self-report depression scale for research in the general population. *Appl. Psychol. Meas.* **1**, 385–401 (1977).

39. Derryberry, D. & Reed, M. A. Anxiety-related attentional biases and their regulation by attentional control. *J. Abnorm. Psychol.* **111**, 225–236 (2002).

40. Patton, J. H., Stanford, M. S. & Barratt, E. S. Factor structure of the Barratt impulsiveness scale. *J. Clin. Psychol.* **51**, 768–774 (1995).

41. Kaufman, E. A. *et al.* The Difficulties in Emotion Regulation Scale Short Form (DERS-SF): Validation and replication in adolescent and adult samples. *J. Psychopathol. Behav. Assess.* **38**, 443–455 (2016).
42. Brown, K. W. & Ryan, R. M. The benefits of being present: Mindfulness and its role in psychological well-being. *J. Pers. Soc. Psychol.* **84**, 822–848 (2003).
43. Steinberg, L. & Monahan, K. C. Age differences in resistance to peer influence. *Dev. Psychol.* **43**, 1531–1543 (2007).
44. Ryff, C. D. Happiness is everything, or is it? Explorations on the meaning of psychological well-being. *J. Pers. Soc. Psychol.* **57**, 1069–1081 (1989).
45. Weinstein, N., Przybylski, A. K. & Ryan, R. M. The index of autonomous functioning: Development of a scale of human autonomy. *J. Res. Personal.* **46**, 397–413 (2012).
46. Russell, D., Peplau, L. A. & Cutrona, C. E. The revised UCLA Loneliness Scale: Concurrent and discriminant validity evidence. *J. Pers. Soc. Psychol.* **39**, 472–480 (1980).
47. Ajzen, I. CONSTRUCTING A THEORY OF PLANNED BEHAVIOR QUESTIONNAIRE.
48. Bot, S. M., Engels, R. C. M. E., Knibbe, R. A. & Meeus, W. H. J. Sociometric Status and Social Drinking: Observations of Modelling and Persuasion in Young Adult Peer Groups. *J. Abnorm. Child Psychol.* **35**, 929–941 (2007).
49. Cosme, D. *et al.* Study protocol: Social Health Impact of Network Effects (SHINE) Study. Preprint at https://doi.org/10.31234/osf.io/cj2nx (2022).
50. Pearson, M. *et al.* Adolescent substance use in different social and peer contexts: A social network analysis. *Drugs Educ. Prev. Policy* **13**, 519–536 (2006).
51. Yarkoni, T., Poldrack, R. A., Nichols, T. E., Van Essen, D. C. & Wager, T. D. Large-scale automated synthesis of human functional neuroimaging data. *Nat. Methods* **8**, 665–670 (2011).
52. Friedman, J. H. Greedy function approximation: A gradient boosting machine. *Ann. Stat.* **29**, 1189–1232 (2001).

53. Lewis, M. A. *et al.* They Drink How Much and Where? Normative Perceptions by Drinking Contexts and Their Association to College Students'Alcohol Consumption. *J. Stud. Alcohol Drugs* **72**, 844–853 (2011).

54. Yanovitzky, I., Stewart, L. P. & Lederman, L. C. Social Distance, Perceived Drinking by Peers, and Alcohol Use by College Students. *Health Commun.* **19**, 1–10 (2006).
